# Italian *Opuntia ficus-indica* Cladodes as Rich Source of Bioactive Compounds with Health-Promoting Properties

**DOI:** 10.3390/foods7020024

**Published:** 2018-02-18

**Authors:** Gabriele Rocchetti, Marco Pellizzoni, Domenico Montesano, Luigi Lucini

**Affiliations:** 1 Department of Animal Science, Food and Nutrition, Università Cattolica del Sacro Cuore, via Emilia Parmense 84, 29122 Piacenza, Italy; gabriele.rocchetti@unicatt.it; 2 Department for Sustainable Food Process, Università Cattolica del Sacro Cuore, via Emilia Parmense 84, 29122 Piacenza, Italy; marco.pellizzoni@unicatt.it (M.P.); luigi.lucini@unicatt.it (L.L.); 3 Department of Pharmaceutical Sciences, Section of Food Science and Nutrition, University of Perugia, Via San Costanzo 1, 06126 Perugia, Italy

**Keywords:** food profiling, polyphenols, β-polysaccharides, antioxidant activity, *Opuntia ficus-indica*, cladodes

## Abstract

Natural by-products, especially phenolic compounds, are in great demand by the nutra-pharmaceutical and biomedical industries. An analytical study was performed to investigate, for the first time, the presence of antioxidant constituents and the corresponding in vitro antioxidant activity in the extract of cladodes from Ficodindia di San Cono (*Opuntia ficus-indica*) protected designation of origin (PDO). The cladode extracts were analysed for target determination of selected constituents, i.e., β-polysaccharides and total phenolic content. Moreover, the antioxidant activity of hydro-alcoholic extracts was assessed by means of two different methods: α, α-diphenyl-β-picrylhydrazyl (DPPH) free radical scavenging method and ferric reducing antioxidant power (FRAP) assay. An untargeted UHPLC-ESI-QTOF-MS profiling approach was used to depict the phenolic profile of hydro-alcoholic cladode extracts. Interestingly, over 2 g/kg of polyphenols were detected in this matrix, and these compounds were mainly responsible for the antioxidant properties, as shown by the strong correlation between phenolic classes and antioxidant scores. Finally, this study provides basic information on the presence of bioactive compounds and in vitro antioxidant activities in cladode extracts from cactus that might recommend their novel applications at the industrial level in the field of nutraceutical products.

## 1. Introduction

The cactus (genus *Opuntia*, family Cactaceae) is a native plant of the American continent and is commonly found at every latitude although it is better adapted to arid areas. Prickly pear cacti can be found all over the world, with a wide climatic tolerance, being able to proliferate in rainfall regimes of 250 to 1200 mm per annum with very hot summers of over 40 °C, and cold winters with temperatures frequently falling below 0 °C for brief durations [1]. 

Nowadays, there are more than 250 species, distributed in Mediterranean Europe, India, the Middle East and in the American and African countries [2]. More than 90% of cultivated crops of the Italian production are grown in Sicily (southern Italy), representing an important food source [3]. In this regard, “Ficodindia di San Cono” (*Opuntia ficus-indica*) is a protected designation of origin (PDO) product from Sicily characterized by a large-sized fruit, with a weight varying from 105 to 270 g (with 5% tolerance). From a botanical point of view, the fruit presents a green or yellowish-orange colored skin for the “Surfarina” cultivar, whilst the “Sanguigna” and “Muscaredda” cultivars range from green to ruby-red and green to straw-white, respectively. 

The main commercial exploitation of this plant remains the fruit, although the vegetative parts, i.e., cladodes, are generally used as animal feed [4,5], fodder or disposed in landfills, whilst in some countries they are also consumed as plants for human consumption [6]. In fact, not only the fruits but also young cladodes are used as a starting point to realize several consumer goods, such as candy, liqueurs, body lotions, creams, and shampoos [7]. Some authors [8] have calculated also the nutritional value of the Opuntia fruits, placing them between that of lettuce and spinach. 

In recent years, the market has shown considerable interest in a variety of tissues of *Opuntia ficus-indica* since they can be used both in food and pharmaceutical areas [6]. In particular, attention is now focused on the difference between food/dietary supplements and nutraceuticals [9]. Nutraceuticals are closer to pharmaceuticals and can help in fighting some of the major health challenges of the century, such as metabolic syndrome, cardiovascular diseases and hypercholesterolemia [9]. In this regard, they are considered ‘beyond the diet before the drugs’, as pointed out in previous works [10]. 

Cactus cladodes contain high amounts of fiber, including pectin, mucilage, lignin, cellulose and hemicellulose, and generally these substances are able to bring wellbeing to the metabolism of lipids and sugars [11]. In particular, β-polysaccharides (i.e., glucose units linked (1→4)-β (as in cellulose) but interspersed with (1→3)-β-linkages), are characterized by an irregular linkage structure that prevents the formation of a crystalline structure leading to a water-soluble capacity [12]. These polysaccharides are generally classified as soluble dietary fiber, improving glucose control and modulate renal water and sodium handling in type 2 diabetes patients; therefore, the high dietary fiber content of cladodes has the capacity to absorb large amounts of water, forming viscous or gelatinous colloids, and determining the absorption of several kinds of organic molecules [13]. *Opuntia ficus-indica* cladodes can also be considered a rich source of bioactive and functional compounds, which make them an important candidate for the production of health-promoting and functional foods. In this regard, in recent years, the scientific world has paid particular attention to polyphenols as they have shown antioxidant properties in vitro, together with protective effects against cancer, and the ability to cure and prevent cardiovascular disorders, inflammatory and allergic diseases [14]. Cardiovascular disease is one of the major factors responsible for death in industrialized countries [15]; therefore, *Opuntia ficus-indica* cladodes could help in lowering cholesterol levels and in preventing hypercholesterolemia [16]. 

Several studies reported the potential of seeds and peels from *Opuntia* spp. as novel by-products with antioxidant activity and bioactive properties [17,18]. However, although traditionally used as a valuable health supporting nutrient, the vegetative parts of *Opuntia* spp. plants (i.e., the non-edible parts) have been scarcely studied, and nowadays there is a lack of information on their entire chemical and bioactive properties. Furthermore, antioxidant properties of *Opuntia* spp. genus have been described only for few species [19], and very little information is available about cultivars located in Italy, in particular about Ficodindia di San Cono PDO and its comprehensive screening of phenolic compounds. 

Therefore, in this work, β-polysaccharides, total phenolic content, antioxidant activity (as FRAP reducing power and DPPH radical scavenging), and the untargeted phenolic profile of extracts from fully-grown cladodes from Ficodindia di San Cono PDO were deeply investigated. The use of an untargeted ultra-high-pressure liquid chromatography coupled to quadrupole-time-of-flight mass spectrometer (UHPLC-ESI-QTOF-MS) system is expected to provide a much deeper investigation of the actual phenolic composition of these cladodes, as compared to classical targeted approaches. Moreover, the obtained fingerprint may be useful to better understand the nutraceutical traits of this species, considering both products and by-products obtainable from the processing of cladodes. 

## 2. Materials and Methods

### 2.1. Cladodes Harvest and Preparation

*Opuntia ficus-indica* cladodes with an approximate length of 25–30 cm were manually harvested from the PDO consortium area, comprising the municipalities of San Cono, San Michele di Ganzaria, Piazza Armerina and Mazzarino (Catania and Enna, Sicily, Italy–altitude: from 200 to 400 m a.s.l.). A sample per municipality (except the site of San Cono, where 2 samples were produced), was prepared by pooling 5 cladodes. In more detail, one apical, three central and one basal position fresh cactus cladodes were washed, and the thorns were removed manually. Then, they were individually cut into small pieces, peeled and homogenized by a mixer, obtaining five samples (replicates). The mean value for each target analyses has been obtained calculating the average among the five leaves. All samples were store at -18°C in freezer until further analysis.

### 2.2. Proximate Composition of Cladodes

The proximate composition analyses were carried out according to AOAC [20] for dry matter (DM; method 930.15), protein (method 976.05), ash (method 942.05) and lipid (method 954.02 without acid hydrolysis), while total carbohydrates were calculated by difference. Samples were also analysed for contents of different fiber fractions, i.e., neutral detergent fiber (NDF), acid detergent fiber (ADF) and acid detergent lignin (ADL), according to the methods described by Van Soest et al. [21]. Finally, the results were expressed as grams of each compound per 100 g of cladodes.

### 2.3. Determination of β-polysaccharides Content

The β-polysaccharides content was determined colorimetrically, recording the absorbance at 540 nm, after reaction with Congo red dye, according to Eberendu et al. [22]. The spectrophotometric measurements were performed using a Perkin Elmer lambda 12 U*V/V*IS spectrophotometer (Ontario, Canada). The samples were extracted by 4 g of inner parenchyma in 10 mL of double distilled sterile water, homogenized and finally shaken using a horizontal shaker for 2 h. After that, 500 μL of 15 g/L KOH together with 2 mL of Congo red solutions (obtained by diluting a saturated aqueous solution 50 times) were added. The final reaction volume was left for 1 h prior the colorimetric reading at λ = 540 nm. In order to perform quantitative determinations, a pure β-glucan standard was used, and the respective results were expressed as β-glucan equivalents. Finally, measurements on five different cladode extracts from *Opuntia ficus-indica* di San Cono were performed. 

### 2.4. Extraction and Profiling of Phenolic Compounds from Cladodes

Each replicate (five) was extracted from 4 g of representative sample of inner parenchyma in 20 mL of 0.1% formic acid in 80:20 (*v/v*) methanol/water (LCMS grade, VWR, Milan, Italy) using an Ultra-turrax (Ika T25, Staufen, Germany) at 25,000 rpm for 3 min. The extracts were then centrifuged at 313 *× g* for 15 min at 4 °C. The resulting solutions were filtered using 0.22 μm cellulose syringe filters and collected in an amber vial for further use. 

The total phenolic contents were investigated by using the Folin-Ciocalteu spectrophotometric assay, as previously reported [23], with small modifications. Aliquots of the sample (1 mL) were mixed with 2.5 mL of Folin-Ciocalteu reagent (Sigma, diluted five-fold) and 4 mL (75 g/L) sodium carbonate. Subsequently, the absorbance was recorded at 765 nm, after 40 min at 20 °C in dark conditions. Finally, a calibration curve was prepared in order to express final results; in particular, aliquots of gallic acid methanolic solutions were prepared to his purpose, and the results expressed as gallic acid equivalents (GAE). 

After that, phenolic compounds were profiled through ultra-high-pressure liquid chromatography (a 1290 liquid chromatographic system equipped with binary pump, from Agilent Technologies, Santa Clara, CA, USA) coupled to a hybrid quadrupole-time-of-flight mass spectrometer (a G6550 mass spectrometer detector, from Agilent Technologies, Santa Clara, CA, USA), by using a JetStream dual electrospray as ionization source (UHPLC-ESI-QTOF-MS). UHPLC-ESI-QTOF-MS analytical conditions were set up on the basis of a previous experiment [24]. Briefly, the acquisition range of mass spectrometer was set from 50 to 1000 *m/z* with the instrument working in positive MS-only mode. In order to keep the mass axis calibrated, lock masses (121.0509 and 922.0098 *m/z*) were continuously infused during analyses using a separate electrospray. A reverse phase C18 column (Agilent Zorbax eclipse plus C18, 50 × 2.1 mm, 1.8 μm) with a water/methanol gradient elution was used for chromatographic separation. In particular, the LC mobile phase A consisted of water (Milli-Q grade, Millipore, Bedford, MA, USA), while the mobile phase B was methanol (LCMS grade, VWR, Milan, Italy). Formic acid 0.1% (*v/v*) and ammonium formate (5 mM) (both from Sigma) were added to both mobile phases. The gradient was initiated with 5% B and increased to 90% B within 15 min, then held for 3 min. The LC mobile phase temperature was set to 35 °C, with a flow rate of 220 μL/min. Injection volume was 3 μL, and source conditions were the following: nitrogen was used as both sheath gas (10 L/min and 350 °C) and drying gas (8 L/min and 330 °C); nebulizer pressure was 60 psig, nozzle voltage was 300 V, and capillary voltage was 3.5 kV. Mass spectrometry raw data were analysed using the Agilent Profinder B.07 software, by using the ‘find-by-formula’ algorithm. In particular, compound identification was realized based on the Phenol-Explorer 3.6 database [25], and using the entire isotopic profile (monoisotopic accurate mass, isotope spacing and ratio) to increase the accuracy. An overall identification score above 85% and a mass accuracy below 5 ppm were adopted as minimum identifications criteria. The mass and retention time alignment with the compounds filtering (post-acquisition data processing) were conducted in Profinder B.07: only those compounds identified within 100% of the replications in at least one cladode sample were considered.

Furthermore, after a classification of polyphenols into phenolic classes and subclasses, cumulative intensities were calculated, and then quantitative values were determined by using methanolic standard solutions of pure phenolics (provided from Extrasyntese, Lyon, France) in order to provide more information. Particularly, the standards used were: matairesinol and sesamin (for dibenzylbutyrolactone and dihydroxydibenzylbutane, and furan and furofuran lignans, respectively), ferulic acid (for hydroxycinnamic acids and other phenolic acids), cyanidin (as representative of anthocyanins subclass), catechin (flavanols), luteolin (flavones and other remaining flavonoids), resveratrol (stilbenes), pentadecylresorcinol or cardol (for alkylresorcinols) and tyrosol (as representative of tyrosols and other remaining phenolics). Therefore, a calibration curve (not weighed and not forced to origin) was built and used for the quantization of phenolic compounds, considering five concentrations of standard over 5 orders of magnitude.

### 2.5. Assay of DPPH Radical Scavenging Activity

The radical scavenging ability of polyphenols against the stable radical DPPH was investigated by using the DPPH spectrophotometric assay, as previously described [23]. Briefly, 2 mL of each phenolic extract solution was placed in a cuvette together with 2 mL of a 1.0 × 10^−4^ mol/L daily-prepared ethanol solution of DPPH. The absorbance measurements were performed at 517 nm using a Perkin Elmer lambda 12 UV/VIS spectrophotometer (Ontario, Canada). The decrease in absorbance was determined continuously after the addition of the DPPH radical at five minute intervals. DPPH radical scavenging activity coefficients were calculated using gallic acid as reference compound, and results were finally expressed as gallic acid equivalents (GAE).

### 2.6. Assay of FRAP Reducing Antioxidant Power

The FRAP assay was carried out on the basis of the colorimetric method previously described by Benzie and Strain [26], with minor modification. In particular, the clinical auto-analyzer ILAB 600 (Instrumentation Laboratory, Lexington, MA, USA) was used to carry out the measurements. The FRAP working reagent was prepared daily by mixing a pH 3.6 acetate buffer 300 mM, a TPTZ (2,4,6-tripyridyl-*s*-triazine) 10 mM in 40 mM HCl solution and FeCl_3_ 20 mM, in the ratio of 10:1:1. The phenolic extracts (100 μL) were mixed with 3000 μL of FRAP working reagent and the absorbance was measured at 600 nm, after 343 s of incubation at 37 °C. A calibration curve was built using gallic acid in ethanol before any session of analysis. Finally, the results were expressed as gallic acid equivalents (GAE). 

### 2.7. Statistical Analysis

Data analysis were carried out with PASW Statistics 25.0 (SPSS Inc.: Chicago, IL, USA). Correlations among β-polysaccharides, total phenolic content, antioxidant activities and phenolic classes equivalents of *Opuntia* cladode samples were obtained through Pearson’s correlation coefficient (*p <* 0.01, two tailed). The elaboration of UHPLC-ESI-QTOF-MS data on phenolic profile of cladodes phenolic extracts were made using Agilent Mass Profiler Professional B.12.06 (Agilent Technologies, Santa Clara, CA, USA). Compounds’ abundance was normalized at the 75th percentile and baselined to their median across all replicates. 

## 3. Results and Discussion

### 3.1. Nutritional Composition, Total Phenolics and β-polysaccharides Contents

The nutritional composition of Ficodindia di San Cono cladodes is presented in Table 1. 

These results showed that San Cono cladodes possessed low protein and lipid content, being 0.58 and 0.12 g/100 g, respectively. De Santiago et al. [27] reported a higher protein content (1.1 g/100 g) and a similar lipid content (<0.1 g/100 g) in fresh cactus cladodes (*Opuntia ficus-indica*) from Spain. San Cono cladodes were particularly abundant in fiber, being the NDF content of 3.42 g/100, and this finding fitted with results obtained by De Santiago et al. [27] and Guevara-Figueroa et al. [28]. Stintzing and Carle [7] reviewed the chemistry and technological use of Opuntia cladodes, showed that, on a fresh weight basis, the typical chemical composition of these matrices is characterized by 3–7 g carbohydrates, 1–2 g minerals, 0.5–1 g proteins, 0.2 g lipids and 1 g of fiber. However, the fact that cladode composition varies according to edaphic factors at the cultivation site, the season and the age of the plant should be taken into account; at this regard, young cladodes show higher carbohydrate, protein and water contents [7]. 

As regards polyphenols, these compounds are widely distributed in the plant kingdom and in recent years they have attracted much attention, due to their in vitro antioxidant capacity with potential beneficial implications in human health [29]. The total phenolic content (TPC), as assayed through the Folin-Ciocalteu approach, along with β-polysaccharides content and in vitro antioxidant activity (DPPH radical scavenging and FRAP reducing power) are reported in Table 2. 

Overall, present findings showed that cladodes from Ficodindia di San Condo PDO possessed considerable nutritional value with health-promoting properties. It has been well studied that the phenolic content of plant materials is strongly correlated with their antioxidant activity [30]. Normally, lipophilic and hydrophilic compounds, such as carotenoids and polyphenols, contribute to the total in vitro antioxidant activity of fruits and vegetables [31,32]. In particular, phenolic compounds are the principal plant constituents with antioxidant properties, which exhibit an important function in neutralizing free radicals [33]. In the present study, the TPC in cladodes of Ficodindia di San Cono PDO was found around 2600 mg GAE/kg fresh weight (FW). This value was comparable to findings reported by De Santiago et al. [27] which obtained a GAE value of 1700 mg/kg FW for cactus cladodes (*Opuntia ficus-indica*) collected from Spain. Other works reported definitely lower TPC values; for example, Santos-Zea et al. [34] reported GAE values of 318 mg/kg DM for cladode flours, considering the variety ‘Jalpa’ of *Opuntia ficus-indica*, whilst other varieties of *Opuntia* spp. showed on average GAE values around 700 mg/kg DM. Furthermore, Ramirez-Moreno et al. [35] determined the levels of polyphenols in cladodes of two species (*Opuntia ficus-indica*, cultivars ‘Milpa’ and ‘Atlixco’), showing GAE values of 5710 and 3820 mg/kg DM, respectively. However, it is important to underline that these differences in TPC could arise above all from different climatic conditions [36]. In particular, looking at *Opuntia* spp. plants, all parts of the cactus are particularly rich in polyphenolic classes, such as various flavonoids and phenolic acids, as reviewed by El-Mostafa et al. [37]. However, the secondary metabolite accumulation in the plant depends on both biotic and abiotic factors. Since the Opuntia species used in this study were cultivated under the same environmental conditions (according to PDO product specifications), the amount and profile of polyphenols were characteristics of *Opuntia* spp. grown in this geographic area. 

Dietary fiber (DF) is considered a combination of chemically heterogeneous substances. Nowadays, the soluble/insoluble DF ratio is an important nutritional parameter, like TDF content, because of the different physiological and beneficial effects [38]. In particular, soluble dietary fiber (SDF) is characterized by compounds with high water holding activity, which can be considered health promoting substrates of intestinal and colonic microbiota. Some studies outlined that the majority component of cladode samples was DF [13]. In this study, the SDF content in cladodes was investigated colorimetrically, showing a value of 2617.39 mg β-glucan equivalents/kg FW. This value was even higher than the β-polysaccharides content of inner gel parenchyma from leaves of two different Aloe species, being on average 828 mg β-glucan equivalents/kg FW [39]. β-glucans, like some other β-polysaccharides, are considered the principal component of the soluble fiber in whole oats and barley, and their biological activities are strongly influenced by the molecular weight. Among cereals, the highest content of β-glucans (as g per 100 g dry weight) has been reported for barley 2–20 g and oats 3–8 g. The other cereals contain these compounds in much lower amounts, with the following values: sorghum 1.1–6.2 g, rye 1.3–2.7 g, maize 0.8–1.7 g, triticale 0.3–1.2 g, wheat 0.5–1.0 g, durum wheat 0.5–0.6 g, and rice 0.13 g [40]. Other studied sources of β-glucans include some types of seaweed and different species of mushrooms [40]. Cladodes from Ficodindia di San Cono PDO were then characterized by approximately 0.3 g/100 g DM of β-glucan equivalents, a value comparable to triticale, rice, and wheat. Therefore, the combination of high DF and associated phytochemicals (such as phenolic compounds) in a single matrix (such as cladodes) results in a food system with specific health related properties, suitable for different uses.

### 3.2. In vitro Antioxidant Activity of Cladodes

The in vitro antioxidant activity should not be determined based on a single antioxidant test [41]. Therefore, in the current work, the in vitro antioxidant activity of samples was evaluated by employing FRAP and DPPH assays, since the aforementioned methods are based on two different reaction mechanisms and kinetics [41]. Furthermore, the DPPH assay is currently considered a valid colorimetric method for the evaluation of antioxidant potential of plant extracts [42]. Furthermore, the literature presents a lack of uniformity in the standards used for calibration or regarding the best antioxidant assay performed; therefore, sometimes the comparison of spectrophotometric results with literature data becomes difficult. 

Antioxidants are a group of compounds very different in chemistries and properties, therefore. the choice of assay could have a great effect upon the results obtained [43]. These antioxidant compounds may help to reduce the oxidative stress, preventing free radicals from damaging biomolecules such as proteins, DNA and lipids [44]. As shown in Table 2, San Cono Opuntia cladodes possessed GAE values of 1040 mg/kg FW considering DPPH radical scavenging, and 1638 mg/kg FW for FRAP reducing power. De Santiago et al. [27] obtained DPPH values in *Opuntia* spp. cladode extracts of 45.05 mg Trolox Equivalents/kg FW, while Astello-Garcia et al. [45] reported on average a DPPH value of 127,000 Trolox Equivalents/kg DW considering three different cultivars of the same species (*Opuntia ficus-indica*). However, these data regarding DPPH were not properly comparable due to the different expression of results. Considering FRAP reducing power of San Cono cladodes, the value obtained (962 μmol GAE/100 g) is higher than those reported by Rocchetti et al. [24], evaluating the antioxidant potential of common gluten-free flours containing polyphenols. Furthermore, cladode extracts also showed higher DPPH radical scavenging values than pumpkin and poppy seeds, being respectively 620 and 860 mg GAE/kg, as reported by Ghisoni et al. [46]. 

### 3.3. Evaluation of Phenolic Profile by UHPLC-ESI-QTOF-MS

**Figure 1:**
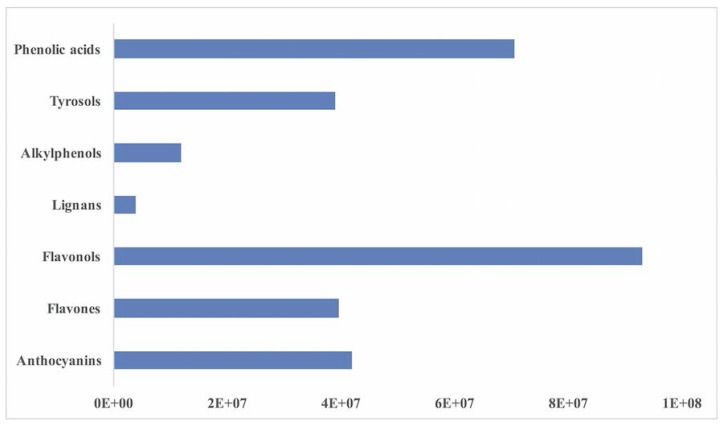
Abundance of different chemical classes of polyphenols in Ficodindia di San Cono PDO cladodes analysed (cumulative intensities as gained from UHPLC-ESI-QTOF-MS profiling).

An untargeted UHPLC-ESI/QTOF-MS approach was used to investigate the entire phenolic profile in cladodes extracts. Flavonoids were definitely the most frequent class of polyphenols detected, with 89 compounds annotated, followed by phenolic acids (54 compounds), tyrosols equivalents (27 compounds), and few other phenolics (lignans, alkylphenols and stilbenes derivatives). The phenolic profile of cladode extracts is shown in Figure 1 considering the cumulative intensity per phenolic class (as gained from UHPLC-ESI/QTOF-MS screening). 

Flavonols showed the highest intensity when compared to other phenolic subclasses, with approximately a difference of one order of magnitude. The entire list of phenolic compounds identified in cladodes extracts, together with annotations (raw formula, identification score, composite mass spectrum), is provided as supplementary material. Even though each compound possessed a different response factor at the electrospray ionization (ESI), the cumulative intensity of phenolic compounds in cladode extracts (when referred to semi-quantitative values) is generally in agreement with the Folin-Ciocalteu assay [24]. Nonetheless, results were elaborated and expressed as equivalents for the main phenolic classes. In particular, as shown in Table 3, cladodes from San Cono PDO were very abundant in anthocyanins (1443.76 mg/kg cyanidin equivalents) and phenolic acids, expressed as ferulic acid equivalents, being 1453.84 mg/kg when referring to the sum of hydroxybenzoic, hydroxyphenylpropanoic and hydroxycinnamic acids. Glycosidic forms of kaempferol (a flavonol) were very abundant, being 241 mg/kg equivalents, together with alkylphenols equivalents which were strongly represented in this matrix being 65.04 mg/kg, followed by flavones equivalents (in particular glycosidic forms of apigenin). Lower values were obtained for both subclasses of lignans (furofurans and  dibenzylbutyrolactones), being 6.52 and 3.47 mg/kg equivalents, respectively. 

Looking at the great abundance in anthocyanins and phenolic acids, the health benefits of these two classes of compounds must be emphasized. In particular, anthocyanins are natural pigments in the plant kingdom, mainly responsible for the blue, purple, red, and orange colors of many fruits and vegetables. In the last year, the interest towards their absorption, metabolism, bioavailability and pharmacokinetics, has increased along with methods for their analytical determination. The interest to deepen the knowledge of the health benefits of anthocyanins has also strongly increased thanks to their anticancer, anti-inflammatory, antidiabetic, and neuroprotective activities together with prevention of cardiovascular diseases [47]. Concerning phenolic acids, they are usually divided into two classes: equivalents of benzoic acid and equivalents of cinnamic acid. The hydroxybenzoic acid content of plants is generally low when compared to hydroxycinnamic acids, with the exception of certain red fruits, black radish and onions [48]. The free form of hydroxycinnamic acids is rapidly absorbed by the small intestine after ingestion. Nevertheless, these compounds are naturally esterified in plant matrices, establishing chemical bonds with this latter, and esterification is able to reduce their absorption due to the lack of enzymes (esterases) able to hydrolyse phenolic acids in the intestinal mucosa, liver and plasma. The hydrolysis can be performed only by the microflora present into the colon; therefore, the polyphenols reaching the colon could promote an antioxidant environment after their hydrolysis into aglycones by bacterial microflora [47]. Regarding San Cono PDO cladodes, phenolic extracts from this plant matrix could provide relevant quantities of these antioxidant and functional compounds.

Overall, present findings fitted with Astello-Garcia et al. [45], which identified phenolic compounds in cladode extracts considering *Opuntia* spp. cultivars with different domestication gradient. In particular, the phenolic profile showed major and minor compounds that were present only in wild or domesticated species. Among polyphenols, the most represented were conjugated forms of isorhamnetin, kaempferol, and quercetin, all belonging to the phenolic class of flavonoids. In another work, Guevara-Figueroa et al. [28] investigated the phenolic composition of commercial and wild leaves from *Opuntia* spp., showing that phenolic acids and flavonoids were the most represented compounds. In particular, these authors identified among phenolic acids ferulic, caffeic, gallic, coumaric, and 2-hydroxybenozoic (also known as salicylic acid) acids. Considering the flavonoids class, conjugated forms of quercetin (rutin and iso-quercitrin), kampferol-3-O-rutinoside (nicotiflorin) and two different forms of narcissin (isorhamnetin derivatives) were detected and quantified in different commercial and wild cladodes. The same findings were shown by El-Mostafa et al. [37], reviewing the possible use of cactus leaves as a source of bioactive compounds. Looking at semi-quantitative values, this work could be considered one of the first identifying anthocyanins as representative  phenolic compounds in cladodes extracts from *Opuntia* spp. In particular, our findings showed that conjugated forms of cyanidin, pelargonidin and petunidin were very abundant in cladodes extracts with average values of 428.27, 161.39 and 114.08 mg/kg equivalents for cyanidin 3,5-*O*-diglucoside, pelargonidin 3-*O*-(6″-malonyl-glucoside) and petunidin 3-*O-*rutinoside,  respectively (supplementary material). Finally, looking at Pearson’s correlations, there were highly significant correlations between TPC and DPPH (*p <* 0.01), whilst flavones (luteolin equivalents) were well correlated to phenolic acids (ferulate equivalents) and flavanols (catechin equivalents) (*p <* 0.05). Interestingly, ferulate and luteolin equivalents were those phenolic compounds better explaining the DPPH radical scavenging activity (*p <* 0.01). However, all the correlation values recorded are shown in Table 4. 

This work can be considered as the first assessing the entire phenolic profile and composition in cladodes of Ficondindia di San Cono PDO thanks to an untargeted UHPLC-ESI/QTOF mass spectrometry approach. The further study on the comparison of phenolic profile of different Opuntia species using a metabolomics-based approach (UHPLC-ESI/QTOF-MS) appears to be worthwhile, in order to select the best source of antioxidant compounds within this plant genus for industrial applications.

## 4. Conclusions

The demand for natural antioxidants, to be used for applications such as nutraceuticals, biopharmaceuticals, as well as food additives, is increasing due to consumers’ preference. In this work, a new source of antioxidant compounds from Ficodindia di San Cono PDO cladodes extracts has been deeply investigated describing its entire phenolic profile. Very high antioxidant activity values have been observed in the hydro-alcoholic extracts of Ficodindia di San Cono cladodes. A high correlation between total phenolic content and DPPH radical scavenging was observed (*p <* 0.01). Moreover, an elevated quantity of β-polysaccharides, included in the group of water-soluble fiber, was accounted. The UHPLC-ESI/QTOF-MS phenolic profiling allowed for the identification of the main phenolic classes and subclasses in cladode extracts, showing that they are good source of equivalent for anthocyanins and phenolic acids, followed by other phenolics. Overall, considering the results obtained, it would seem possible to use cactus cladodes as a source of natural and antioxidant compounds, possibly incorporating them into foods, cosmetics or pharmaceutical products. From a health-promoting perspective, these cladode extracts could be considered as new and very promising sources of natural antioxidants. Our findings provide a basis for developing a valuable food additive, based on *Opuntia ficus-indica* cladodes from San Cono (Sicily, Italy), thanks to their water-soluble fiber, phenolic composition and the related antioxidant activity.

## Figures and Tables

**Table 1:** Chemical composition (on a fresh weight basis) of Ficodindia di San Cono cladodes. Data are presented as mean values ± standard deviation (*n* = 5). NDF = neutral detergent fiber; ADF = acid detergent fiber; ADL = acid detergent lignin. * = obtained by difference.

Parameters	San Cono cladodes
Moisture (g water/100 g cladodes)	92.33 ± 1.36
Protein (%)	0.58 ± 0.02
Ash (%)	0.50 ± 0.01
Lipid (%)	0.12 ± 0.02
Carbohydrates (%) *	3.05
NDF (%)	3.42 ± 0.63
ADF (%)	0.83 ± 0.14
ADL (%)	0.12 ± 0.02

**Table 2:** Total β-polysaccharides, total phenolic content (TPC), and antioxidant activities (DPPH radical scavenging and FRAP reducing power) in cladodes extracts. The data are presented as mean values ± standard deviation (*n =* 5). Results for TPC, DPPH and FRAP are expressed as gallic acid equivalents (GAE), whilst results for β-polysaccharides are expressed as β-glucan equivalents. FW = fresh weight.

	*β-Polysaccharides*	*TPC*	*DPPH*	*FRAP*
	*(mg β-Glucan Equivalents/kg FW)*	*(mg GAE/kg FW)*	*(mg GAE/kg FW)*	*(mg GAE/kg FW)*
**Cladodes**	2617.39 ± 225.58	2633.10 ± 214.78	1040.03 ± 112.42	1638.17 ± 41.30

**Table 3:** Quantifications per classes of phenolics identified from untargeted UHPLC-ESI-QTOF-MS in Ficodindia di San Cono PDO cladodes. Results are expressed in mg/kg of phenolic equivalents. The data are presented as mean values ± standard deviation (*n =* 3). Glu = glucoside.

Phenolic Class	Phenolic Derivatives	mg/kg Equivalents
*Flavonoids—Anthocyanins*	Cyanidin-Glu	1058.55 ± 10.49
	Pelargonidin-Glu	187.97 ± 10.42
	Petunidin-Glu	186.55 ± 3.11
	Delphinidin-Glu	2.81 ± 0.31
	Malvidin-Glu	4.31 ± 0.08
*Flavonoids—Flavones*	Luteolin-Glu	5.14 ± 1.59
	Apigenin-Glu	40.69 ± 0.58
	Isoflavonoids	6.81 ± 0.52
*Flavonoids—Flavonols*	Myricetin-Glu	8.52 ± 0.55
	Quercetin-Glu	8.97 ± 0.69
	Kaempferol-Glu	241.68 ± 3.39
	Isorhamnetin-Glu	98.42 ± 8.52
*Lignans*	Furofurans	6.52 ± 2.24
	Dibenzylbutyrolactone	3.47 ± 0.02
*Other phenolics*	Alkylphenols	65.04 ± 10.43
	Hydroxybenzaldehydes	0.43 ± 0.06
	Hydroxycoumarins	7.81 ± 0.52
	Tyrosols	12.89 ± 1.02
*Phenolic acids*	Hydroxybenzoics	114.01 ± 3.34
	Hydroxyphenylpropanoics	91.58 ± 5.98
	Hydroxycinnamics	1248.24 ± 103.15

**Table 4:** Pearson’s correlations coefficients between total phenolic content (TPC), DPPH radical scavenging, FRAP reducing power and phenolic subclasses equivalents. ^**^ = *p <* 0.01; ^*^ = *p <* 0.05; n.s. = not significant.

	DPPH	FRAP	TPC	Cyanidin Eq.	Luteolin Eq.	Catechin Eq.	Ferulate Eq.	Matairesinol Eq.	Tyrosol Eq.	Cardol Eq.
DPPH	1	n.s.	1 ^**^	0.84 ^*^	0.95 ^**^	0.92 ^*^	0.96 ^**^	n.s.	n.s.	0.59
FRAP	n.s.	1	n.s.	−0.74	n.s.	n.s.	n.s.	0.90	−0.84	−0.61
TPC	1 ^**^	n.s.	1	−0.84	0.95	−0.92	0.96 ^**^	n.s.	n.s.	0.59
Cyanidin Eq.	0.84 ^*^	−0.74	−0.84	1	−0.64	0.56	−0.65	n.s.	n.s.	n.s.
Luteolin Eq.	0.95 ^**^	n.s.	0.95	−0.64	1	−0.99 ^*^	1 ^*^	−0.45	0.56	0.80
Catechin Eq.	0.92 ^*^	n.s.	−0.92	0.56	−0.99 ^*^	1	−0.99	0.54	−0.64	−0.86
Ferulate Eq.	0.96 ^**^	n.s.	0.96 ^**^	−0.65	1 ^*^	−0.99	1	−0.44	0.54	0.79
Matairesinol Eq.	n.s.	0.90	n.s.	n.s.	−0.45	0.54	−0.44	1	−0.99	−0.89
Tyrosol Eq.	n.s.	−0.84	n.s.	n.s.	0.56	−0.64	0.54	−0.99	1	0.94
Cardol Eq.	0.59	−0.61	0.59	n.s.	0.80	−0.86	0.79	−0.89	0.94	1

## Supplementary Materials

The following are available online at http://www.mdpi.com/2304-8158/7/2/24/s1. Table S1: Whole dataset of phenolic compounds identified in cladode extracts, together with relative abundances and annotations (raw formula, identification score, composite spectrum).
