# Supplementary material for: Italian Opuntia ficus-indica Cladodes as Rich Source of Bioactive Compounds with Health-Promoting Properties
Source: Foods. 2018 Feb 18;7(2):24. doi: 10.3390/foods7020024 (PMC5848128; doi:10.3390/foods7020024)
Supplement: Supplementary file 1 [file foods-07-00024-s001.pdf]

Compound	Subclass	Formula (DB)	Area	Height	Base Peak	End	Ions	Polarity	Mass	Score	m/z	RT
Cyanidin	Anthocyanins	C15 H11 O6	364140	60121	122,0812	9,485	2	Positive	287,0556	85,44	287,055	9,327
Cyanidin 3,5-O-diglucoside	Anthocyanins	C27 H31 O16	12380583	1997109	633,1433	9,842	9	Positive	611,1588	99,59	611,161	9,525
Cyanidin 3,5-O-diglucoside	Anthocyanins	C27 H31 O16	552908	63998	498,2603	9,386	5	Positive	611,1599	94,16	611,1605	9,267
Cyanidin 3-O-glucosyl-rutinoside	Anthocyanins	C33 H41 O20	15763531	2232682	498,2604	9,624	10	Positive	757,2184	98,02	757,2199	9,228
Cyanidin 3-O-sambubiosyl 5-O-glucoside	Anthocyanins	C32 H39 O20	819928	146538	122,0813	8,02	6	Positive	743,2008	98,12	743,2022	7,882
Cyanidin 3-O-xylosyl-rutinoside	Anthocyanins	C32 H39 O19	616059	104998	498,2602	9,248	6	Positive	727,2061	98,59	727,2078	9,129
Delphinidin 3-O-galactoside	Anthocyanins	C21 H21 O12	73741	19548	793,2173	9,366	3	Positive	465,1013	79,74	465,102	9,307
Malvidin 3-O-(6''-caffeoyl-glucoside)	Anthocyanins	C32 H31 O15	124325	20152	122,0814	12,871	2	Positive	655,1659	73,24	655,1654	12,752
Pelargonidin	Anthocyanins	C15 H11 O5	87330	15315	122,0813	15,424	2	Positive	271,061	75,96	271,0604	15,345
Pelargonidin 3,5-O-diglucoside	Anthocyanins	C27 H31 O15	464267	79017	512,276	10,792	5	Positive	595,1633	95,26	595,1655	10,633
Pelargonidin 3,5-O-diglucoside	Anthocyanins	C27 H31 O15	88740	13302	498,2603	9,228	2	Positive	595,1666	73,86	595,1659	9,109
Pelargonidin 3-O-(6''-malonyl-glucoside)	Anthocyanins	C24 H23 O13	4746437	653982	512,276	11,128	4	Positive	519,1116	88,4	501,1006	10,653
Peonidin	Anthocyanins	C16 H13 O6	100859	16029	122,0813	15,207	2	Positive	301,0713	85,16	301,0707	15,108
Petunidin 3-O-galactoside	Anthocyanins	C22 H23 O12	937456	145630	647,159	11,029	5	Positive	479,1183	99,74	479,1184	10,891

Petunidin 3-O-galactoside	Anthocyanins	C22 H23 O12	1159225	208814	633,1433	9,782	4	Positive	479,1187	99,33	479,1182	9,525
Petunidin 3-O-rutinoside	Anthocyanins	C28 H33 O16	3293453	418411	498,2604	9,406	4	Positive	625,1769	99,79	625,1765	9,208
Dihydromyricetin 3-O-rhamnoside	Dihydroflavonols	C21 H22 O12	156320	21789	122,0814	10,356	3	Positive	466,1108	78,73	471,0896	10,198
Dihydroquercetin	Dihydroflavonols	C15 H12 O7	364140	60121	122,0812	9,485	3	Positive	304,058	85,44	287,055	9,327
Dihydroquercetin 3-O-rhamnoside	Dihydroflavonols	C21 H22 O11	132691	20553	122,0813	3,585	3	Positive	450,118	88,24	473,1075	3,486
(-)-Epigallocatechin 3-O-gallate	Flavanols	C22 H18 O11	104877	19021	104,1081	1,289	5	Positive	458,0849	96,87	463,0635	1,091
Eriodictyol	Flavanones	C15 H12 O6	84804	14565	122,0813	15,424	2	Positive	288,0637	75,96	271,0604	15,325
Hesperetin	Flavanones	C16 H14 O6	549654	94156	122,0813	17,721	3	Positive	302,0791	99,19	285,0757	17,523
Naringin 4'-O-glucoside	Flavanones	C33 H42 O19	201434	42847	379,1002	5,248	4	Positive	742,232	74,16	765,2201	5,149
6-Hydroxyluteolin	Flavones	C15 H10 O7	306553	23170	498,2603	9,406	3	Positive	302,0426	81,53	303,0499	9,307
6-Hydroxyluteolin	Flavones	C15 H10 O7	631412	48177	122,0813	8,238	2	Positive	302,0425	81,92	303,0498	8
7,3',4'-Trihydroxyflavone	Flavones	C15 H10 O5	84804	14565	122,0813	15,424	2	Positive	270,0531	75,96	271,0604	15,325
Apigenin 6-C-glucoside	Flavones	C21 H20 O10	186759	29902	122,0814	11,603	2	Positive	432,1054	81,61	455,0947	11,465
Apigenin 7-O-(6''-malonyl-apiosyl-glucoside)	Flavones	C29 H30 O17	27375926	4165053	633,1433	9,861	5	Positive	650,1465	93,84	633,1433	9,525
Chrysoeriol 7-O-(6''-malonyl-apiosyl-glucoside)	Flavones	C30 H32 O18	1558868	257956	122,0814	8,911	4	Positive	680,1561	89,06	663,1529	8,693

Apigenin 6,8-di-C-glucoside	Flavones	C27 H30 O15	102583	18014	122,0814	11,188	3	Positive	594,1579	75,27	617,1474	11,069
Apigenin 6,8-di-C-glucoside	Flavones	C27 H30 O15	1216583	195444	512,276	10,851	6	Positive	594,1583	99,29	617,1476	10,633
Apigenin 6,8-di-C-glucoside	Flavones	C27 H30 O15	211870	36269	122,0814	10,237	4	Positive	594,1583	95,24	617,1473	10,099
Apigenin 6,8-di-C-glucoside	Flavones	C27 H30 O15	109496	15654	498,2603	9,228	3	Positive	594,1586	73,86	595,1659	9,149
Cirsilineol	Flavones	C18 H16 O7	1688492	150284	104,1072	1,606	5	Positive	344,0906	74,49	326,08	1,249
Cirsimaritin	Flavones	C17 H14 O6	117196	17511	122,0813	17,82	3	Positive	314,0794	80,01	297,076	17,622
Diosmin	Flavones	C28 H32 O15	214045	34193	122,0813	7,842	3	Positive	608,174	93,87	631,163	7,723
Biochanin A	Isoflavonoids	C16 H12 O5	549654	94156	122,0813	17,721	3	Positive	284,0685	99,19	285,0757	17,523
Luteolin 7-O-(2-apiosyl-glucoside)	Flavones	C26 H28 O15	94387	17324	512,276	10,713	3	Positive	580,1422	73,91	603,1315	10,614
Luteolin 7-O-(2-apiosyl-glucoside)	Flavones	C26 H28 O15	1650130	274386	122,0813	9,525	6	Positive	580,1427	99,81	603,132	9,327
3,7-Dimethylquercetin	Flavonols	C17 H14 O7	380822	53120	122,0813	3,071	4	Positive	330,074	82,88	353,0634	2,972
6,8-Dihydroxykaempferol	Flavonols	C15 H12 O8	178952	23170	498,2603	9,406	3	Positive	320,0532	85,32	303,0498	9,307
Isorhamnetin	Flavonols	C16 H12 O7	3770503	597412	647,159	11,188	4	Positive	316,0585	99,41	317,0658	10,891
Isorhamnetin	Flavonols	C16 H12 O7	8459500	1026234	498,2604	9,426	4	Positive	316,0587	99,12	317,066	9,208
Isorhamnetin 3-O-galactoside	Flavonols	C22 H22 O12	5143059	614144	512,276	11,128	7	Positive	478,1112	99,67	479,1185	10,653

Isorhamnetin 3-O-galactoside	Flavonols	C22 H22 O12	1012507	170007	633,1433	9,782	4	Positive	478,1109	99,33	479,1182	9,525
Isorhamnetin 3-O-galactoside	Flavonols	C22 H22 O12	671938	89324	498,2604	9,406	3	Positive	478,1107	96,93	479,118	9,208
Isorhamnetin 3-O-glucoside 7-O-rhamnoside	Flavonols	C28 H32 O16	2406153	412039	512,2761	10,772	7	Positive	624,169	99,62	647,1585	10,713
Isorhamnetin 3-O-glucoside 7-O-rhamnoside	Flavonols	C28 H32 O16	3122074	395386	498,2604	9,406	4	Positive	624,1691	99,79	625,1765	9,208
Kaempferide	Flavonols	C16 H12 O6	98327	16029	122,0813	15,207	2	Positive	300,0635	85,16	301,0707	15,108
Kaempferol	Flavonols	C15 H10 O6	141130	21511	512,276	10,772	2	Positive	286,0477	83,77	287,055	10,633
Kaempferol	Flavonols	C15 H10 O6	364140	60121	122,0812	9,485	2	Positive	286,0478	85,44	287,055	9,327
Kaempferol	Flavonols	C15 H10 O6	373548	43759	498,2603	9,228	2	Positive	286,0477	84,9	287,0549	9,109
Kaempferol 3-O-(2''-rhamnosyl-6''-acetyl-galactoside) 7-O-rhamnoside	Flavonols	C35 H42 O20	278167	47465	122,0814	12,653	3	Positive	782,2289	94,03	787,2076	12,534
Kaempferol 3-O-(2''-rhamnosyl-galactoside) 7-O-rhamnoside	Flavonols	C33 H40 O19	475553	81065	498,2603	9,228	6	Positive	740,2149	92,83	741,2238	9,089
Kaempferol 3-O-(2''-rhamnosyl-galactoside) 7-O-rhamnoside	Flavonols	C33 H40 O19	562999	96855	122,0813	9,03	5	Positive	740,2151	81,34	763,2053	8,931
6-Hydroxyluteolin 7-O-rhamnoside	Flavones	C21 H20 O11	208714	24943	512,276	10,851	3	Positive	448,1003	71,79	471,0895	10,614
6-Hydroxyluteolin 7-O-rhamnoside	Flavones	C21 H20 O11	156320	21789	122,0814	10,356	3	Positive	448,1003	78,73	471,0896	10,198
Kaempferol 3-O-glucosyl-rhamnosyl-galactoside	Flavonols	C33 H40 O20	25246677	3426728	498,2604	9,564	10	Positive	756,2124	98,11	757,2198	9,228
Kaempferol 3-O-glucosyl-rhamnosyl-galactoside	Flavonols	C33 H40 O20	423366	74860	122,0813	8,238	5	Positive	756,2107	93,68	757,2184	8,099

Kaempferol 3-O-glucosyl-rhamnosyl-galactoside	Flavonols	C33 H40 O20	407055	72184	122,0813	8,02	5	Positive	756,2104	92,37	779,2001	7,921
Kaempferol 3,7-O-diglucoside	Flavonols	C27 H30 O16	31809807	4945402	633,1433	9,842	9	Positive	610,1539	99,72	611,1608	9,525
Kaempferol 3,7-O-diglucoside	Flavonols	C27 H30 O16	785139	106755	498,2603	9,386	6	Positive	610,1532	98,69	633,1421	9,267
Kaempferol 3-O-xylosyl-rutinoside	Flavonols	C32 H38 O19	1168338	203048	498,2602	9,287	7	Positive	726,2005	99,67	749,1898	9,129
Myricetin 3-O-rhamnoside	Flavonols	C21 H20 O12	239979	34258	122,0812	9,505	4	Positive	464,0949	80,35	487,0842	9,307
Myricetin 3-O-rhamnoside	Flavonols	C21 H20 O12	710205	119061	498,2603	9,228	5	Positive	464,0951	99,19	487,0843	9,069
Myricetin 3-O-rutinoside	Flavonols	C27 H30 O17	86207	16155	122,0813	6,535	2	Positive	626,1472	70,29	649,1364	6,436
Quercetin 3-O-arabinoside	Flavonols	C20 H18 O11	373161	61863	122,0813	8,951	3	Positive	434,0843	81,67	439,0634	8,812
Quercetin 3-O-glucosyl-xyloside	Flavonols	C26 H28 O11	116082	12851	122,0812	3,031	3	Positive	516,1626	74,35	521,1416	2,912
Quercetin 3-O-xylosyl-rutinoside	Flavonols	C32 H38 O20	1514109	258241	122,0813	8,06	7	Positive	742,1952	99,16	765,1846	7,882
Quercetin 4'-O-glucoside	Flavonols	C21 H20 O7	131819	18400	498,2603	9,215	3	Positive	384,1199	96,74	366,1088	9,149
Quercetin 7,4'-O-diglucoside	Flavonols	C27 H30 O12	218674	14716	122,0813	3,467	4	Positive	546,173	74,07	551,1524	3,13
6''-O-Acetyldaidzin	Isoflavonoids	C23 H22 O10	70836	12714	122,0814	8,713	3	Positive	458,1218	87,08	481,111	8,614
6''-O-Acetylgenistin	Isoflavonoids	C23 H22 O11	116192	20690	122,0812	6,06	4	Positive	474,1158	98,7	497,1052	5,961
6''-O-Acetylglycitin	Isoflavonoids	C24 H24 O11	80840	13519	633,1433	9,644	2	Positive	488,1299	70,68	489,1369	9,545

6''-O-Malonylgenistin	Isoflavonoids	C24 H22 O13	4036996	653982	512,276	10,772	4	Positive	518,1038	88,62	501,1006	10,653
1-Acetoxypinoresinol	Lignans	C22 H24 O8	430595	39755	104,1079	1,428	7	Positive	416,1469	88,85	399,143	1,15
Cyclolariciresinol	Lignans	C20 H24 O6	188923	13956	293,2116	21,087	2	Positive	360,1568	83,66	343,1535	20,948
Dimethylmatairesinol	Lignans	C22 H26 O6	2942311	510004	122,0809	17,404	9	Positive	386,1727	99,18	387,18	17,167
Medioresinol	Lignans	C21 H24 O7	145949	28260	349,0897	4,912	4	Positive	388,1521	78,11	371,1474	4,853
Syringaresinol	Lignans	C22 H26 O8	362835	63782	122,0813	8,214	7	Positive	418,1629	99,02	401,1598	8,099
4-Vinylsyringol	Alkylmethoxyphenols	C10 H12 O3	785920	55908	365,106	21,279	2	Positive	180,0786	87,72	163,0753	20,948
4-Vinylsyringol	Alkylmethoxyphenols	C10 H12 O3	164650	26150	122,0813	3,104	2	Positive	180,0785	73,54	181,0862	2,992
3-Methylcatechol	Alkylphenols	C7 H8 O2	1440489	233037	263,053	5,301	3	Positive	124,0523	97,61	107,0489	5,031
3-Methylcatechol	Alkylphenols	C7 H8 O2	730579	94571	365,1059	2,469	4	Positive	124,0525	87,38	107,0492	2,378
3-Methylcatechol	Alkylphenols	C7 H8 O2	933260	38141	268,1044	2,219	4	Positive	124,0523	96,43	107,0491	2,16
4-Ethylphenol	Alkylphenols	C8 H10 O	2939994	483571	122,0814	3,566	3	Positive	122,0734	97,97	105,07	3,209
4-Vinylphenol	Alkylphenols	C8 H8 O	1702360	356392	166,0866	2,477	3	Positive	120,0575	87,78	103,0543	2,417
5-Heneicosylresorcinol	Alkylphenols	C27 H48 O2	1574244	27840	738,664	32,668	2	Positive	404,3649	79,95	409,3435	31,243
5-Nonadecenylresorcinol	Alkylphenols	C25 H42 O2	2523718	17831	738,6639	33,816	6	Positive	374,3185	87,69	357,3149	30,886
p-Anisaldehyde	Hydroxybenzaldehydes	C8 H8 O2	805328	99942	349,0897	5,209	2	Positive	136,0523	87,68	119,049	4,853

Vanillin	Hydroxybenzaldehydes	C8 H8 O3	108027	22960	122,0813	6,903	4	Positive	152,0474	72,47	153,0548	6,813
2,3-Dihydroxy-1-guaiacylpropanone	Hydroxybenzoketones	C10 H12 O5	775731	135804	379,1002	5,347	3	Positive	212,0684	99,1	195,0651	5,169
3-Methoxyacetophenone	Hydroxybenzoketones	C9 H10 O2	1346476	140358	122,0813	2,477	4	Positive	150,068	96,09	151,0753	2,239
3-Methoxyacetophenone	Hydroxybenzoketones	C9 H10 O2	9001446	891711	268,1043	2,101	4	Positive	150,0678	99,48	151,0751	1,863
Sinapaldehyde	Hydroxycinnamaldehydes	C11 H12 O4	698443	95713	122,0813	7,208	3	Positive	208,0737	86,67	191,0703	6,852
Sinapaldehyde	Hydroxycinnamaldehydes	C11 H12 O4	226644	41466	122,0813	5,774	3	Positive	208,0736	86,97	191,0702	5,644
4-Hydroxycoumarin	Hydroxycoumarins	C9 H6 O3	1762692	173388	738,6634	29,758	3	Positive	162,0317	99,76	163,039	29,204
4-Hydroxycoumarin	Hydroxycoumarins	C9 H6 O3	1641060	259200	149,0237	21,482	3	Positive	162,0318	99,57	163,039	21,324
Esculin	Hydroxycoumarins	C15 H16 O9	948160	39878	268,1046	2,396	3	Positive	340,0794	83,21	363,0685	1,982
Mellein	Hydroxycoumarins	C10 H10 O3	883110	148494	263,053	5,169	4	Positive	178,0626	89,06	161,0594	5,031
1,4-Naphtoquinone	Naphtoquinones	C10 H6 O2	30329	6362	263,053	5,09	2	Positive	158,037	77,59	159,0438	5,031
3,4-Dihydroxyphenylglycol	Other polyphenols	C8 H10 O4	114150	20879	122,0813	6,892	3	Positive	170,0576	72,99	153,0548	6,813
Arbutin	Other polyphenols	C12 H16 O7	341707	62275	122,0814	7,365	3	Positive	272,0898	85,75	277,0685	7,268
Phlorin	Other polyphenols	C12 H16 O8	8534401	1179792	104,1081	1,566	7	Positive	288,0848	79,83	289,0921	1,091
Pyrogallol	Other polyphenols	C6 H6 O3	2532029	400225	104,1078	1,467	7	Positive	126,0321	97,23	127,0386	1,091

Carnosol	Phenolic terpenes	C20 H26 O4	204898	24092	365,106	20,79	2	Positive	330,1823	81,69	331,1896	20,671
Carvacrol	Phenolic terpenes	C10 H14 O	3470223	26102	738,6638	32,629	5	Positive	150,1047	85,7	133,1011	30,075
Hydroxytyrosol 4-O-glucoside	Tyrosols	C14 H20 O9	1645214	273179	122,0813	6,971	3	Positive	332,1111	92,33	337,0895	6,813
Ligstroside-aglycone	Tyrosols	C19 H21 O7	131819	18400	498,2603	9,215	3	Positive	361,1301	93,61	366,1088	9,149
Oleoside 11-methylester	Tyrosols	C17 H24 O11	1489223	212409	122,0813	6,753	4	Positive	404,1318	99,79	409,1105	6,496
Oleoside dimethylester	Tyrosols	C18 H26 O11	412480	72989	122,0812	6,039	5	Positive	418,1472	98,57	441,1361	5,941
2-Hydroxybenzoic acid	Hydroxybenzoic acids	C7 H6 O3	236325	35274	738,6632	29,544	3	Positive	138,0318	82,11	121,0285	29,204
2-Hydroxybenzoic acid	Hydroxybenzoic acids	C7 H6 O3	462447	61457	149,0237	21,733	2	Positive	138,0318	84,99	121,0285	21,562
2-Hydroxybenzoic acid	Hydroxybenzoic acids	C7 H6 O3	633511	96943	149,0237	21,463	2	Positive	138,0318	87,76	121,0285	21,324
2-Hydroxybenzoic acid	Hydroxybenzoic acids	C7 H6 O3	144844	26252	122,0807	2,75	3	Positive	138,032	76,71	139,0391	2,714
4-Hydroxybenzoic acid 4-O-glucoside	Hydroxybenzoic acids	C13 H16 O8	1641933	218941	122,0808	3,071	5	Positive	300,084	98,61	323,0731	2,695
5-O-Galloylquinic acid	Hydroxybenzoic acids	C14 H16 O10	493536	62396	349,0897	5,011	3	Positive	344,0744	97,45	367,0638	4,853
Benzoic acid	Hydroxybenzoic acids	C7 H6 O2	456330	54919	122,0813	6,991	3	Positive	122,0368	86,89	105,0334	6,694
Gallic acid ethyl ester	Hydroxybenzoic acids	C9 H10 O5	520483	46148	365,106	22,096	2	Positive	198,053	83,88	181,0497	21,562
Gallic acid ethyl ester	Hydroxybenzoic acids	C9 H10 O5	482804	68102	149,0237	21,463	2	Positive	198,053	87,25	181,0497	21,324
Protocatechuic acid 4-O-glucoside	Hydroxybenzoic acids	C13 H16 O9	310722	58437	104,1075	1,289	2	Positive	316,0802	83,48	321,0589	1,15

1,2-Diferuloylgentiobiose	Hydroxycinnamic acids	C32 H38 O17	284397	52408	122,0813	9,505	3	Positive	694,2104	96,49	717,1994	9,406
24-Methylcholestanol ferulate	Hydroxycinnamic acids	C38 H58 O4	452427	71485	738,6619	27,362	7	Positive	578,435	91,48	601,4223	27,184
24-Methylcholesterol ferulate	Hydroxycinnamic acids	C38 H56 O4	177047	14777	365,106	22,294	5	Positive	576,4159	84,78	599,4082	21,958
3,4-Diferuloylquinic acid	Hydroxycinnamic acids	C27 H28 O12	5038532	103088	122,0812	4,457	3	Positive	544,1608	76,55	527,1575	2,338
1-Caffeoylquinic acid	Hydroxycinnamic acids	C16 H18 O9	227871	22945	122,0808	2,794	3	Positive	354,0947	70,61	355,102	2,497
3-Feruloylquinic acid	Hydroxycinnamic acids	C17 H20 O9	558182	73138	122,0813	6,238	3	Positive	368,1108	84,92	391,1003	5,922
3-Sinapoylquinic acid	Hydroxycinnamic acids	C18 H22 O10	92293	16210	122,0813	7,268	3	Positive	398,1227	82,99	421,1105	7,149
3-p-Coumaroylquinic acid	Hydroxycinnamic acids	C16 H18 O8	132718	17624	122,0814	12,633	2	Positive	338,1001	71,53	339,1074	12,376
3-p-Coumaroylquinic acid	Hydroxycinnamic acids	C16 H18 O8	605925	82068	513,1582	6,615	4	Positive	338,1001	98,36	339,1071	6,258
3-p-Coumaroylquinic acid	Hydroxycinnamic acids	C16 H18 O8	236166	29579	122,0812	5,585	2	Positive	338,1003	72,84	339,1076	5,169
3-p-Coumaroylquinic acid	Hydroxycinnamic acids	C16 H18 O8	834472	71675	104,1068	1,626	3	Positive	338,1002	80,94	321,0966	1,348
5-5'-Dehydrodiferulic acid	Hydroxycinnamic acids	C20 H18 O8	91198	16065	122,0813	3,625	3	Positive	386,0999	76,5	369,0968	3,526
Caffeoyl glucose	Hydroxycinnamic acids	C15 H18 O9	213106	36786	122,0812	4,485	4	Positive	342,0939	76,32	343,1008	4,338
Caffeoyl glucose	Hydroxycinnamic acids	C15 H18 O9	80787	18900	166,0866	2,475	2	Positive	342,0945	76,52	343,1022	2,457
Caffeoyl tartaric acid	Hydroxycinnamic acids	C13 H12 O9	171284	26117	104,1075	1,302	3	Positive	312,0486	75,73	335,0377	1,131
Cinnamic acid	Hydroxycinnamic acids	C9 H8 O2	212557	36964	122,0812	18,691	2	Positive	148,0518	82,09	149,0592	18,632

Feruloyl glucose	Hydroxycinnamic acids	C16 H20 O9	3262812	459509	122,0813	6,08	6	Positive	356,1107	99,74	379,0999	5,763
Feruloyl glucose	Hydroxycinnamic acids	C16 H20 O9	12835893	2038854	379,1002	5,545	7	Positive	356,1108	99,22	379,1002	5,189
Hydroxycaffeic acid	Hydroxycinnamic acids	C9 H8 O5	4373739	583664	104,1082	1,665	3	Positive	196,0373	77,37	219,027	1,071
Isoferulic acid	Hydroxycinnamic acids	C10 H10 O4	1218398	128547	122,0813	15,741	3	Positive	194,058	99,59	177,0547	15,246
Isoferulic acid	Hydroxycinnamic acids	C10 H10 O4	2970860	405949	263,053	5,506	7	Positive	194,0578	99,72	177,0546	5,169
m-Coumaric acid	Hydroxycinnamic acids	C9 H8 O3	292405	35326	104,1069	1,487	4	Positive	164,0471	72,88	147,0439	1,329
p-Coumaric acid 4-O-glucoside	Hydroxycinnamic acids	C15 H18 O8	19814472	2795498	349,0898	5,209	5	Positive	326,1005	99,19	349,0898	4,853
p-Coumaric acid 4-O-glucoside	Hydroxycinnamic acids	C15 H18 O8	3576963	264592	104,1072	1,863	6	Positive	326,1014	77,68	308,0909	1,249
p-Coumaric acid ethyl ester	Hydroxycinnamic acids	C11 H12 O3	229256	44157	263,053	5,086	2	Positive	192,0787	85,35	193,0859	4,991
Rosmarinic acid	Hydroxycinnamic acids	C18 H16 O8	5605913	726375	349,0898	5,07	5	Positive	360,0847	80,4	365,064	4,853
Sinapic acid	Hydroxycinnamic acids	C11 H12 O5	342437	44026	122,0813	6,678	3	Positive	224,0684	87,1	207,0651	6,476
Sitostanyl ferulate	Hydroxycinnamic acids	C39 H60 O5	261579	33692	738,6639	30,547	4	Positive	608,4421	78,8	590,4294	30,51
Stigmastanol ferulate	Hydroxycinnamic acids	C39 H60 O4	118330	11764	738,6629	28,293	3	Positive	592,4514	73,49	615,4405	28,214
3,4-dihydroxyphenyl-2-oxypropanoic acid	Hydroxyphenylpropanoic acids	C9 H8 O4	1100938	163922	738,6632	29,494	4	Positive	180,0423	99,7	163,039	29,204

3,4-dihydroxyphenyl-2-oxypropanoic acid	Hydroxyphenyl propanoic acids	C9 H8 O4	2092705	324445	149,0237	21,482	5	Positive	180,0424	99,57	163,039	21,324
Dihydro-p-coumaric acid	Hydroxyphenyl propanoic acids	C9 H10 O3	212557	36964	122,0812	18,691	2	Positive	166,0623	82,09	149,0592	18,632
Dihydro-p-coumaric acid	Hydroxyphenyl propanoic acids	C9 H10 O3	179731	44575	166,0868	2,477	2	Positive	166,0627	77,02	149,0597	2,417
Dihydrocaffeic acid	Hydroxyphenyl propanoic acids	C9 H10 O4	524041	85100	349,0898	5,031	3	Positive	182,0579	87,53	165,0545	4,853
d-Viniferin	Stilbenes	C28 H22 O6	56780	10965	122,0813	8,772	2	Positive	454,1414	72,54	436,1294	8,693
Cyanidin	Anthocyanins	C15 H11 O6	267777	43667	498,2604	9,21	2	Positive	287,0555	85,85	287,0549	9,111
Cyanidin 3,5-O-diglucoside	Anthocyanins	C27 H31 O16	12405550	1990624	633,1435	9,883	8	Positive	611,1589	99,18	611,1612	9,507
Cyanidin 3,5-O-diglucoside	Anthocyanins	C27 H31 O16	585836	64811	498,2604	9,368	5	Positive	611,1601	93,6	611,1606	9,249
Cyanidin 3-O-(6''-acetyl-galactoside)	Anthocyanins	C23 H23 O12	148988	23662	122,0813	3,627	3	Positive	491,1184	82,09	473,1077	3,508
Cyanidin 3-O-glucosyl-rutinoside	Anthocyanins	C33 H41 O20	16207071	2365763	498,2603	9,606	10	Positive	757,2182	98,59	757,2196	9,21
Cyanidin 3-O-sambubiosyl 5-O-glucoside	Anthocyanins	C32 H39 O20	828128	141635	122,0813	8,002	6	Positive	743,2009	97,95	743,2022	7,863
Cyanidin 3-O-xylosyl-rutinoside	Anthocyanins	C32 H39 O19	614942	110475	498,2604	9,229	6	Positive	727,206	98,71	727,2076	9,111
Delphinidin 3-O-galactoside	Anthocyanins	C21 H21 O12	78941	14698	122,0813	7,23	3	Positive	465,1011	70,9	465,1003	7,131

Malvidin 3-O-(6''-caffeoyl-glucoside)	Anthocyanins	C32 H31 O15	128024	19191	122,0813	12,872	2	Positive	655,1657	72,65	655,1651	12,734
Pelargonidin 3,5-O-diglucoside	Anthocyanins	C27 H31 O15	89667	14276	498,2605	9,21	2	Positive	595,1661	70,87	595,1657	9,091
Pelargonidin 3-O-(6''-malonyl-glucoside)	Anthocyanins	C24 H23 O13	4620250	621294	512,276	11,09	4	Positive	519,1115	88,03	501,1005	10,635
Pelargonidin 3-O-galactoside	Anthocyanins	C21 H21 O10	309505	29668	268,1047	1,825	3	Positive	433,1118	75,55	415,0997	1,607
Pelargonidin 3-O-galactoside	Anthocyanins	C21 H21 O10	170341	32250	104,1071	1,489	3	Positive	433,1114	84,42	415,1006	1,469
Peonidin	Anthocyanins	C16 H13 O6	96748	17723	122,0813	15,188	2	Positive	301,0712	75,16	301,0706	15,089
Petunidin 3-O-galactoside	Anthocyanins	C22 H23 O12	929339	142040	647,1592	11,011	5	Positive	479,1181	99,73	479,1184	10,853
Petunidin 3-O-galactoside	Anthocyanins	C22 H23 O12	1215910	203559	633,1435	9,744	4	Positive	479,1188	99,55	479,1184	9,507
Petunidin 3-O-rutinoside	Anthocyanins	C28 H33 O16	3365883	435971	498,2603	9,388	4	Positive	625,1769	99,81	625,1765	9,19
3-Hydroxyphloretin 2'-O-xylosyl-glucoside	Dihydrochalcones	C26 H32 O15	50646	13344	122,0813	6,692	2	Positive	584,1736	75,53	585,1811	6,656
Dihydromyricetin 3-O-rhamnoside	Dihydroflavonols	C21 H22 O12	117462	19841	122,0814	10,298	3	Positive	466,1106	78,32	471,0893	10,18
Dihydroquercetin	Dihydroflavonols	C15 H12 O7	242036	41500	498,2604	9,21	3	Positive	304,0579	85,85	287,0549	9,111
Dihydroquercetin 3-O-rhamnoside	Dihydroflavonols	C21 H22 O11	99887	18803	122,0813	3,583	3	Positive	450,1178	81,98	473,1073	3,528
(-)-Epigallocatechin 3-O-gallate	Flavanols	C22 H18 O11	72843	16315	104,1081	1,211	4	Positive	458,0848	92,45	463,0634	1,073
Hesperetin	Flavanones	C16 H14 O6	507979	86217	122,0813	17,742	3	Positive	302,079	99,61	285,0757	17,525
Naringin 4'-O-glucoside	Flavanones	C33 H42 O19	199421	43918	379,1002	5,23	4	Positive	742,2323	75,54	765,2205	5,151

6-Hydroxyluteolin	Flavones	C15 H10 O7	259522	20989	498,2604	9,395	3	Positive	302,0427	84,83	303,05	9,249
6-Hydroxyluteolin	Flavones	C15 H10 O7	594272	49810	122,0813	8,207	2	Positive	302,0426	81,4	303,0499	7,982
Apigenin 6-C-glucoside	Flavones	C21 H20 O10	156118	25618	122,0813	11,575	2	Positive	432,1054	81,31	455,0945	11,447
Apigenin 6-C-glucoside	Flavones	C21 H20 O10	65724	15520	513,1583	6,312	2	Positive	432,107	77,71	455,0962	6,24
Apigenin 6-C-glucoside	Flavones	C21 H20 O10	116982	30733	104,1071	1,488	3	Positive	432,1035	81,93	415,1005	1,469
Apigenin 7-O-(6''-malonyl-apiosyl-glucoside)	Flavones	C29 H30 O17	28100701	4265580	633,1435	10,002	5	Positive	650,1467	94,74	633,1435	9,507
Apigenin 7-O-apiosyl-glucoside	Flavones	C26 H28 O14	87149	20060	104,1081	1,211	6	Positive	564,1483	71,25	569,1277	1,112
Apigenin 7-O-glucuronide	Flavones	C21 H18 O11	48621	10529	122,0813	7,14	2	Positive	446,0847	70,84	451,0633	7,052
Chrysoeriol 7-O-(6''-malonyl-apiosyl-glucoside)	Flavones	C30 H32 O18	1528832	264690	122,0813	8,862	4	Positive	680,1564	90,48	663,1532	8,675
Apigenin 6,8-di-C-glucoside	Flavones	C27 H30 O15	75517	16277	122,0813	11,14	3	Positive	594,1583	73,78	617,1474	11,051
Apigenin 6,8-di-C-glucoside	Flavones	C27 H30 O15	1125562	185089	512,276	10,803	6	Positive	594,1582	99,18	617,1475	10,615
Apigenin 6,8-di-C-glucoside	Flavones	C27 H30 O15	181461	31213	122,0814	10,194	4	Positive	594,1581	92,32	617,1472	10,101
Apigenin 6,8-di-C-glucoside	Flavones	C27 H30 O15	87538	13599	498,2605	9,205	3	Positive	594,1583	70,87	595,1657	9,13
Cirsimaritin	Flavones	C17 H14 O6	85487	15374	122,0813	17,809	3	Positive	314,0793	84,95	297,0759	17,624
Diosmin	Flavones	C28 H32 O15	193291	33024	122,0813	7,815	3	Positive	608,174	94,75	631,1631	7,705

Biochanin A	Isoflavonoids	C16 H12 O5	477687	84701	122,0813	17,729	3	Positive	284,0684	99,64	285,0757	17,525
Luteolin 7-O-(2-apiosyl-glucoside)	Flavones	C26 H28 O15	1693375	281585	122,0813	9,496	6	Positive	580,1426	99,35	603,1318	9,309
3,7-Dimethylquercetin	Flavonols	C17 H14 O7	397214	53404	122,0813	3,092	4	Positive	330,0742	83,35	353,0636	2,993
6,8-Dihydroxykaempferol	Flavonols	C15 H12 O8	300437	22505	498,2604	9,408	3	Positive	320,0533	85,11	303,05	9,249
Isorhamnetin	Flavonols	C16 H12 O7	7320005	1237560	633,1435	9,843	4	Positive	316,0587	98,74	317,066	9,507
Isorhamnetin	Flavonols	C16 H12 O7	8186513	991745	498,2603	9,408	4	Positive	316,0586	99,15	317,0659	9,19
Isorhamnetin 3-O-galactoside	Flavonols	C22 H22 O12	4990032	581451	512,276	11,09	7	Positive	478,1112	99,61	479,1185	10,655
Isorhamnetin 3-O-galactoside	Flavonols	C22 H22 O12	985908	165075	633,1435	9,744	4	Positive	478,111	99,55	479,1184	9,507
Isorhamnetin 3-O-galactoside	Flavonols	C22 H22 O12	700326	87313	498,2603	9,388	3	Positive	478,1108	96,76	479,1182	9,19
Isorhamnetin 3-O-glucoside 7-O-rhamnoside	Flavonols	C28 H32 O16	2190255	369038	512,276	10,754	7	Positive	624,1689	99,54	647,1583	10,694
Isorhamnetin 3-O-glucoside 7-O-rhamnoside	Flavonols	C28 H32 O16	3211871	408723	498,2603	9,388	4	Positive	624,1691	99,81	625,1765	9,19
Kaempferide	Flavonols	C16 H12 O6	96748	17723	122,0813	15,188	2	Positive	300,0634	75,16	301,0706	15,089
Kaempferol	Flavonols	C15 H10 O6	358302	59182	122,0813	9,487	2	Positive	286,0477	85,63	287,0549	9,309
Kaempferol	Flavonols	C15 H10 O6	267777	43667	498,2604	9,21	2	Positive	286,0477	85,85	287,0549	9,111
Kaempferol	Flavonols	C15 H10 O6	106432	19106	122,0813	9,012	2	Positive	286,0479	83,86	287,0551	8,913

Kaempferol 3-O-(2''-rhamnosyl-6''-acetyl-galactoside) 7-O-rhamnoside	Flavonols	C35 H42 O20	416679	67580	122,0813	12,872	3	Positive	782,2286	94,68	787,2072	12,734
Kaempferol 3-O-(2''-rhamnosyl-galactoside) 7-O-rhamnoside	Flavonols	C33 H40 O19	467917	78662	498,2605	9,21	6	Positive	740,2144	92,87	741,223	9,071
Kaempferol 3-O-(2''-rhamnosyl-galactoside) 7-O-rhamnoside	Flavonols	C33 H40 O19	557626	99014	122,0813	9,012	6	Positive	740,2151	82,03	763,2053	8,913
Kaempferol 3-O-acetyl-glucoside	Flavonols	C23 H22 O12	137990	20970	122,0813	3,627	3	Positive	490,1106	82,09	473,1077	3,528
6-Hydroxyluteolin 7-O-rhamnoside	Flavones	C21 H20 O11	187819	21739	512,276	10,833	5	Positive	448,1004	71,93	471,0893	10,615
6-Hydroxyluteolin 7-O-rhamnoside	Flavones	C21 H20 O11	156236	22008	122,0813	10,378	3	Positive	448,1	75,03	471,0893	10,18
Kaempferol 3-O-glucosyl-rhamnosyl-galactoside	Flavonols	C33 H40 O20	26067602	3641865	498,2603	9,546	10	Positive	756,2122	98,59	757,2196	9,21
Kaempferol 3-O-glucosyl-rhamnosyl-galactoside	Flavonols	C33 H40 O20	427036	77428	122,0813	8,24	5	Positive	756,2109	86,43	757,2186	8,081
Kaempferol 3-O-glucosyl-rhamnosyl-galactoside	Flavonols	C33 H40 O20	409351	74720	122,0813	8,002	5	Positive	756,2106	96,43	779,2001	7,903
Kaempferol 3,7-O-diglucoside	Flavonols	C27 H30 O16	32527093	5023274	633,1435	10,002	9	Positive	610,1541	99,33	611,1611	9,507
Kaempferol 3,7-O-diglucoside	Flavonols	C27 H30 O16	836996	105361	498,2603	9,368	6	Positive	610,1533	97,97	633,1421	9,249
Kaempferol 3-O-xylosyl-rutinoside	Flavonols	C32 H38 O19	1175877	204281	498,2604	9,269	7	Positive	726,2004	99,19	749,1897	9,111
Myricetin 3-O-rhamnoside	Flavonols	C21 H20 O12	240977	32385	122,0813	9,467	4	Positive	464,0949	79,34	487,084	9,289
Myricetin 3-O-rhamnoside	Flavonols	C21 H20 O12	689621	111324	498,2605	9,19	5	Positive	464,095	99,11	487,0843	9,051
Myricetin 3-O-rhamnoside	Flavonols	C21 H20 O12	78941	14698	122,0813	7,23	3	Positive	464,0933	70,9	465,1003	7,131

Myricetin 3-O-rutinoside	Flavonols	C27 H30 O17	84991	13838	122,0813	6,517	2	Positive	626,1477	74,02	649,137	6,418
Quercetin 3-O-arabinoside	Flavonols	C20 H18 O11	385607	61263	122,0813	8,952	3	Positive	434,0842	82,61	439,0632	8,814
Quercetin 3-O-glucosyl-xyloside	Flavonols	C26 H28 O11	128047	14460	122,0813	3,053	3	Positive	516,1627	82,99	521,1417	2,894
Quercetin 3-O-xylosyl-rutinoside	Flavonols	C32 H38 O20	1481272	244569	122,0813	8,035	7	Positive	742,1952	99,22	765,1845	7,863
Quercetin 4'-O-glucoside	Flavonols	C21 H20 O7	440489	32212	122,0813	8,152	3	Positive	384,1199	96,53	366,1087	7,943
Rhamnetin	Flavonols	C16 H27 O7	36646	5195	336,2285	5,369	2	Positive	331,1759	74,33	313,1632	5,27
6''-O-Acetyldaidzin	Isoflavonoids	C23 H22 O10	68689	12211	122,0814	8,695	3	Positive	458,1217	76,08	481,1106	8,596
6''-O-Acetylgenistin	Isoflavonoids	C23 H22 O11	127557	22298	122,0813	10,061	3	Positive	474,1156	94,66	497,1051	9,962
6''-O-Malonylgenistin	Isoflavonoids	C24 H22 O13	3909390	621294	512,276	10,754	4	Positive	518,1037	88,19	501,1006	10,635
Glycitin	Isoflavonoids	C22 H22 O10	52101	10323	122,0813	9,091	3	Positive	446,122	70,15	451,1001	9,012
1-Acetoxypinoresinol	Lignans	C22 H24 O8	420285	34013	104,1079	1,409	7	Positive	416,1475	86,79	439,1373	1,152
Cyclolariciresinol	Lignans	C20 H24 O6	196962	15719	293,2115	21,108	2	Positive	360,1568	83,4	343,1534	20,95
Dimethylmatairesinol	Lignans	C22 H26 O6	2946071	513546	122,0809	17,386	9	Positive	386,1726	98,56	387,1798	17,168
Medioresinol	Lignans	C21 H24 O7	167710	29990	349,0898	4,933	3	Positive	388,1517	79,42	371,1468	4,854
Syringaresinol	Lignans	C22 H26 O8	366296	63572	122,0813	8,196	7	Positive	418,1629	98,59	401,1596	8,081
4-Vinylsyringol	Alkylmethoxyphenols	C10 H12 O3	808192	57168	365,106	21,222	2	Positive	180,0786	87,48	163,0753	20,93

3-Methylcatechol	Alkylphenols	C7 H8 O2	1304091	212286	263,053	5,312	3	Positive	124,0524	98,11	107,049	5,032
3-Methylcatechol	Alkylphenols	C7 H8 O2	1193045	22691	122,0813	4,221	4	Positive	124,0526	95,27	107,0491	3,29
3-Methylcatechol	Alkylphenols	C7 H8 O2	244173	82176	365,1059	2,395	5	Positive	124,0525	94,47	107,0491	2,379
4-Ethylphenol	Alkylphenols	C8 H10 O	2880789	467082	122,0815	3,508	3	Positive	122,0734	97,3	105,0701	3,211
4-Vinylphenol	Alkylphenols	C8 H8 O	1388753	370654	166,0864	2,478	3	Positive	120,0573	87,61	103,0541	2,439
5-Heneicosylresorcinol	Alkylphenols	C27 H48 O2	384082	19859	738,6643	31,7	2	Positive	404,3656	81,13	409,3443	31,304
5-Nonadecenylresorcinol	Alkylphenols	C25 H42 O2	2599023	16764	738,6641	33,838	5	Positive	374,3186	83,2	357,315	31,264
Demethoxycurcumin	Curcuminoids	C20 H18 O5	12251	20625	365,1058	2,36	4	Positive	338,1159	76,09	343,0924	2,36
p-Anisaldehyde	Hydroxybenzaldehydes	C8 H8 O2	808080	107481	349,0898	5,171	2	Positive	136,0523	87,86	119,049	4,854
Vanillin	Hydroxybenzaldehydes	C8 H8 O3	116470	22475	122,0813	6,904	3	Positive	152,0476	86,73	153,0546	6,794
2,3-Dihydroxy-1-guaiacylpropanone	Hydroxybenzoketones	C10 H12 O5	866211	140583	263,0531	5,324	4	Positive	212,0684	99,57	195,065	5,191
3-Methoxyacetophenone	Hydroxybenzoketones	C9 H10 O2	734166	122507	122,0813	4,617	5	Positive	150,068	97,56	151,0754	4,458
3-Methoxyacetophenone	Hydroxybenzoketones	C9 H10 O2	8982132	908860	268,1044	2,102	4	Positive	150,068	99,8	151,0753	1,865
Sinapaldehyde	Hydroxycinnamaldehydes	C11 H12 O4	232570	31801	149,0236	21,484	4	Positive	208,0741	97,64	209,0812	21,326
Sinapaldehyde	Hydroxycinnamaldehydes	C11 H12 O4	297761	44062	122,0813	6,002	3	Positive	208,0736	86,9	191,0704	5,646
4-Hydroxycoumarin	Hydroxycoumarins	C9 H6 O3	1330086	162440	149,0236	21,9	3	Positive	162,0317	99,72	163,0389	21,583

4-Hydroxycoumarin	Hydroxycoumarins	C9 H6 O3	1662054	246303	149,0236	21,484	3	Positive	162,0317	99,82	163,039	21,326
Esculin	Hydroxycoumarins	C15 H16 O9	927890	38826	268,1045	2,379	3	Positive	340,0795	82,85	363,0686	2,003
Mellein	Hydroxycoumarins	C10 H10 O3	804853	133386	263,053	5,171	4	Positive	178,0626	90,71	161,0594	5,032
Acetyl eugenol	Hydroxyphenylpropenes	C12 H14 O3	1909686	259560	104,1074	1,528	5	Positive	206,0934	84,11	229,0821	1,132
1,4-Naphtoquinone	Naphtoquinones	C10 H6 O2	31754	5755	263,053	5,112	2	Positive	158,0367	74,94	159,0436	5,032
3,4-Dihydroxyphenylglycol	Other polyphenols	C8 H10 O4	123980	22163	122,0813	6,913	3	Positive	170,0576	88,62	153,0546	6,814
Arbutin	Other polyphenols	C12 H16 O7	347370	59425	122,0813	7,385	2	Positive	272,0899	85,83	277,0686	7,269
Phlorin	Other polyphenols	C12 H16 O8	8598372	1174981	104,108	1,588	7	Positive	288,0848	78,88	293,0639	1,093
Pyrogallol	Other polyphenols	C6 H6 O3	565580	112036	365,1033	23,226	3	Positive	126,0319	97,47	149,0211	23,147
Carnosol	Phenolic terpenes	C20 H26 O4	187117	24228	365,106	20,791	2	Positive	330,1824	82,1	331,1895	20,672
Carvacrol	Phenolic terpenes	C10 H14 O	2857764	23649	738,6642	32,452	5	Positive	150,1046	85,56	133,1011	30,136
Hydroxytyrosol 4-O-glucoside	Tyrosols	C14 H20 O9	1627987	271848	122,0814	6,992	5	Positive	332,1112	92,01	337,0895	6,794
Ligstroside-aglycone	Tyrosols	C19 H21 O7	1099644	110483	263,053	5,205	3	Positive	361,1298	93,2	366,1087	5,072
Oleoside 11-methylester	Tyrosols	C17 H24 O11	1894329	313653	122,0813	7,818	5	Positive	404,1318	99,82	409,1104	7,626
Oleoside dimethylester	Tyrosols	C18 H26 O11	441908	69701	122,0813	6,042	4	Positive	418,1471	98,6	441,1362	5,943

2-Hydroxybenzoic acid	Hydroxybenzoic acids	C7 H6 O3	221117	33496	738,664	29,443	3	Positive	138,0318	75,03	121,0285	29,205
2-Hydroxybenzoic acid	Hydroxybenzoic acids	C7 H6 O3	579639	62382	365,106	21,95	2	Positive	138,0319	84,67	121,0286	21,563
2-Hydroxybenzoic acid	Hydroxybenzoic acids	C7 H6 O3	664728	97787	149,0236	21,464	2	Positive	138,0318	86,8	121,0285	21,326
2-Hydroxybenzoic acid	Hydroxybenzoic acids	C7 H6 O3	174077	27569	122,0809	2,838	3	Positive	138,032	76,9	139,0392	2,696
4-Hydroxybenzoic acid 4-O-glucoside	Hydroxybenzoic acids	C13 H16 O8	1612055	217556	122,0807	3,072	4	Positive	300,0841	99,03	323,0733	2,716
5-O-Galloylquinic acid	Hydroxybenzoic acids	C14 H16 O10	492740	65789	349,0898	5,013	2	Positive	344,0745	84,19	367,0637	4,854
Benzoic acid	Hydroxybenzoic acids	C7 H6 O2	437713	50518	122,0813	6,992	3	Positive	122,0368	86,82	105,0334	6,695
Gallic acid ethyl ester	Hydroxybenzoic acids	C9 H10 O5	584523	48313	365,106	22,236	2	Positive	198,053	84,12	181,0497	21,563
Gallic acid ethyl ester	Hydroxybenzoic acids	C9 H10 O5	456299	64793	149,0236	21,464	2	Positive	198,0529	87,15	181,0496	21,326
Protocatechuic acid 4-O-glucoside	Hydroxybenzoic acids	C13 H16 O9	281021	54234	104,1075	1,271	2	Positive	316,0801	80,24	321,0587	1,152
1,2-Diferuloylgentiobiose	Hydroxycinnamic acids	C32 H38 O17	208055	34688	633,1435	9,724	3	Positive	694,2103	96,73	717,1997	9,566
1-Sinapoyl-2-feruloylgentiobiose	Hydroxycinnamic acids	C33 H40 O18	153249	26477	122,0813	8,457	3	Positive	724,2185	78,47	707,2151	8,358
2-S-Glutathionyl caftaric acid	Hydroxycinnamic acids	C23 H27 N3 O15 S	24967	9275	104,1081	1,132	3	Positive	617,1177	81,15	599,1084	1,073
24-Methylcholestanol ferulate	Hydroxycinnamic acids	C38 H58 O4	519489	78253	738,6619	27,483	6	Positive	578,4346	87,61	583,4142	27,186
24-Methylcholesterol ferulate	Hydroxycinnamic acids	C38 H56 O4	170070	13283	365,106	22,316	4	Positive	576,4159	81,64	599,4082	21,979

3,4-Diferuloylquinic acid	Hydroxycinnamic acids	C27 H28 O12	665159	27848	122,0813	4,399	3	Positive	544,1598	81,76	527,1568	4,062
1-Caffeoylquinic acid	Hydroxycinnamic acids	C16 H18 O9	173955	29925	122,0814	2,32	4	Positive	354,0956	79,76	359,0749	2,221
3-Feruloylquinic acid	Hydroxycinnamic acids	C17 H20 O9	171179	21061	122,0813	7,547	3	Positive	368,1119	71,72	373,0893	7,448
3-Feruloylquinic acid	Hydroxycinnamic acids	C17 H20 O9	578685	68526	122,0813	6,26	5	Positive	368,1124	83,13	391,1003	5,923
3-Sinapoylquinic acid	Hydroxycinnamic acids	C18 H22 O10	63446	15386	122,0813	7,235	2	Positive	398,1211	81,78	421,1102	7,151
3-Sinapoylquinic acid	Hydroxycinnamic acids	C18 H22 O10	242386	18689	268,1045	1,805	4	Positive	398,1212	78,71	399,1273	1,508
3-p-Coumaroylquinic acid	Hydroxycinnamic acids	C16 H18 O8	86727	15718	122,0813	12,467	2	Positive	338,1001	83,97	339,1074	12,357
3-p-Coumaroylquinic acid	Hydroxycinnamic acids	C16 H18 O8	573859	80409	513,1583	6,61	4	Positive	338,1002	98,79	339,1072	6,24
3-p-Coumaroylquinic acid	Hydroxycinnamic acids	C16 H18 O8	161105	30583	379,1002	5,369	2	Positive	338,1	81,2	339,1074	5,171
3-p-Coumaroylquinic acid	Hydroxycinnamic acids	C16 H18 O8	923491	74663	104,1069	1,586	3	Positive	338,1008	79,24	321,0972	1,33
Caffeoyl glucose	Hydroxycinnamic acids	C15 H18 O9	210036	36101	122,0813	4,474	5	Positive	342,0943	87,22	343,1009	4,359
Caffeoyl glucose	Hydroxycinnamic acids	C15 H18 O9	43140	18393	365,1058	2,574	2	Positive	342,094	71,55	343,1019	2,498
Caffeoyl tartaric acid	Hydroxycinnamic acids	C13 H12 O9	170139	26434	104,1075	1,305	3	Positive	312,0486	73,57	335,0378	1,132
Cinnamic acid	Hydroxycinnamic acids	C9 H8 O2	202112	38363	122,0812	18,673	2	Positive	148,0518	79,83	149,0592	18,613
Feruloyl glucose	Hydroxycinnamic acids	C16 H20 O9	632220	80884	122,0813	6,359	5	Positive	356,1107	99	339,1072	6,24

Feruloyl glucose	Hydroxycinnamic acids	C16 H20 O9	12939703	2075287	379,1002	5,527	7	Positive	356,1108	99,15	379,1002	5,191
Hydroxycaffeic acid	Hydroxycinnamic acids	C9 H8 O5	4223809	570955	104,1082	1,667	3	Positive	196,0373	77,59	219,0269	1,073
Isoferulic acid	Hydroxycinnamic acids	C10 H10 O4	1129345	130409	122,0813	15,664	3	Positive	194,0581	99,66	177,0547	15,248
Isoferulic acid	Hydroxycinnamic acids	C10 H10 O4	2891240	392940	263,0531	5,448	6	Positive	194,0578	99,57	195,065	5,191
m-Coumaric acid	Hydroxycinnamic acids	C9 H8 O3	302542	32919	104,1068	1,568	4	Positive	164,0469	75,95	165,0547	1,33
p-Coumaric acid 4-O-glucoside	Hydroxycinnamic acids	C15 H18 O8	20083070	2884258	349,0898	5,211	5	Positive	326,1006	98,94	349,0898	4,854
p-Coumaric acid 4-O-glucoside	Hydroxycinnamic acids	C15 H18 O8	161079	27196	122,0812	4,656	2	Positive	326,101	82,79	349,0903	4,577
p-Coumaric acid ethyl ester	Hydroxycinnamic acids	C11 H12 O3	241848	44000	263,053	5,092	2	Positive	192,0787	85,56	193,086	4,993
Rosmarinic acid	Hydroxycinnamic acids	C18 H16 O8	5613804	742746	349,0898	5,092	4	Positive	360,0848	80,34	365,064	4,854
Sinapic acid	Hydroxycinnamic acids	C11 H12 O5	582003	98118	263,0531	5,225	4	Positive	224,0683	86,88	207,065	5,092
Sitosterol ferulate	Hydroxycinnamic acids	C39 H58 O4	320162	90927	738,6639	29,343	6	Positive	590,4363	71,25	590,4329	29,324
Verbascoside	Hydroxycinnamic acids	C29 H36 O15	405876	27053	122,0813	3,251	3	Positive	624,2027	76,51	625,2096	3,132
3,4-dihydroxyphenyl-2-oxypropanoic acid	Hydroxyphenylpropanoic acids	C9 H8 O4	1730606	204908	149,0236	21,999	5	Positive	180,0423	99,72	163,0389	21,563
3,4-dihydroxyphenyl-2-oxypropanoic acid	Hydroxyphenylpropanoic acids	C9 H8 O4	1960890	300597	149,0236	21,484	5	Positive	180,0423	99,82	163,039	21,326

Dihydro-p-coumaric acid	Hydroxyphenyl propanoic acids	C9 H10 O3	202112	38363	122,0812	18,673	2	Positive	166,0623	79,83	149,0592	18,613
Dihydrocaffeic acid	Hydroxyphenyl propanoic acids	C9 H10 O4	543441	86827	349,0898	5,052	2	Positive	182,0578	87,57	165,0545	4,854
Dihydrocaffeic acid	Hydroxyphenyl propanoic acids	C9 H10 O4	211639	25063	104,107	1,469	2	Positive	182,058	75,12	165,0549	1,33
Cyanidin	Anthocyanins	C15 H11 O6	361666	62462	122,0813	9,488	2	Positive	287,0555	85,85	287,0549	9,31
Cyanidin 3,5-O-diglucoside	Anthocyanins	C27 H31 O16	12487732	2029692	633,1434	9,845	8	Positive	611,1589	99,3	611,1611	9,528
Cyanidin 3,5-O-diglucoside	Anthocyanins	C27 H31 O16	538122	64936	498,2603	9,389	5	Positive	611,1602	89,58	611,1607	9,271
Cyanidin 3-O-glucosyl-rutinoside	Anthocyanins	C33 H41 O20	15764134	2256943	498,2603	9,667	10	Positive	757,2184	98,21	757,2198	9,211
Cyanidin 3-O-sambubiosyl 5-O-glucoside	Anthocyanins	C32 H39 O20	820553	139705	122,0813	8,023	6	Positive	743,201	98,46	743,2026	7,885
Cyanidin 3-O-xylosyl-rutinoside	Anthocyanins	C32 H39 O19	601111	100782	498,2603	9,251	6	Positive	727,2058	98,08	727,2075	9,112
Delphinidin 3-O-galactoside	Anthocyanins	C21 H21 O12	91248	18640	122,0813	9,389	3	Positive	465,1017	78,68	465,1022	9,31
Malvidin 3-O-(6''-caffeoyl-glucoside)	Anthocyanins	C32 H31 O15	123584	20892	122,0813	12,854	2	Positive	655,1657	72,44	655,1652	12,735
Pelargonidin 3,5-O-diglucoside	Anthocyanins	C27 H31 O15	442775	75428	512,276	10,775	4	Positive	595,1634	72,1	595,1653	10,617
Pelargonidin 3,5-O-diglucoside	Anthocyanins	C27 H31 O15	87096	14261	498,2603	9,251	2	Positive	595,1659	73,95	595,1654	9,112

Pelargonidin 3-O-(6''-malonyl-glucoside)	Anthocyanins	C24 H23 O13	4680108	667036	512,2759	10,993	4	Positive	519,1116	88,84	501,1006	10,656
Pelargonidin 3-O-galactoside	Anthocyanins	C21 H21 O10	573611	37176	268,1047	1,827	3	Positive	433,1114	74,76	415,0998	1,51
Peonidin	Anthocyanins	C16 H13 O6	111834	17328	122,0813	15,25	3	Positive	301,0713	77,72	301,0708	15,111
Petunidin 3-O-galactoside	Anthocyanins	C22 H23 O12	941859	151461	647,1589	11,033	5	Positive	479,1183	99,73	479,1184	10,874
Petunidin 3-O-galactoside	Anthocyanins	C22 H23 O12	1123775	209839	633,1434	9,746	5	Positive	479,1188	99,67	479,1183	9,528
Petunidin 3-O-rutinoside	Anthocyanins	C28 H33 O16	3269497	420913	498,2603	9,409	4	Positive	625,177	99,8	625,1765	9,211
3-Hydroxyphloretin 2'-O-xylosyl-glucoside	Dihydrochalcones	C26 H32 O15	50323	12327	379,1001	5,212	2	Positive	584,1744	75,35	585,1817	5,133
Dihydromyricetin 3-O-rhamnoside	Dihydroflavonols	C21 H22 O12	190867	22444	512,2759	10,835	3	Positive	466,1107	74,43	471,0893	10,617
Dihydroquercetin	Dihydroflavonols	C15 H12 O7	366284	66349	122,0813	9,488	3	Positive	304,0579	85,85	287,0549	9,31
Dihydroquercetin 3-O-rhamnoside	Dihydroflavonols	C21 H22 O11	180292	29376	122,0814	11,567	2	Positive	450,1161	82,03	455,0948	11,448
(-)-Epigallocatechin 3-O-gallate	Flavanols	C22 H18 O11	97171	20048	104,1081	1,253	4	Positive	458,0846	99,07	463,0635	1,074
Hesperetin	Flavanones	C16 H14 O6	509887	85297	122,0813	17,704	3	Positive	302,0791	99,63	285,0758	17,526
Naringin 4'-O-glucoside	Flavanones	C33 H42 O19	197668	42209	379,1001	5,252	4	Positive	742,2322	73,71	765,2205	5,153
Naringin 6'-malonate	Flavanones	C30 H34 O17	107104	26402	263,0531	5,133	4	Positive	666,1765	79,08	649,173	5,074
Pinocembrin	Flavanones	C15 H12 O4	1066461	75329	268,1046	2,163	3	Positive	256,073	70,4	261,0522	1,55
6-Hydroxyluteolin	Flavones	C15 H10 O7	131768	20350	498,2603	9,403	3	Positive	302,0427	79,48	303,0499	9,271

6-Hydroxyluteolin	Flavones	C15 H10 O7	5639 85	461 41	122, 0813	8,20 6	2	Posi tive	302, 0426	81,4 5	303, 0499	8,00 4
Apigenin 6-C-glucoside	Flavones	C21 H20 O10	1501 05	270 05	122, 0814	11,5 59	2	Posi tive	432, 1056	82,0 3	455, 0948	11,4 48
Apigenin 6-C-glucoside	Flavones	C21 H20 O10	5264 0	129 99	513, 1584	6,29 6	2	Posi tive	432, 1066	79,0 7	455, 0959	6,24 2
Apigenin 7-O-(6''-malonyl-apiosyl-glucoside)	Flavones	C29 H30 O17	2786 4214	424 353 8	633, 1433	9,82 5	5	Posi tive	650, 1465	93,7 3	633, 1433	9,52 8
Apigenin 7-O-apiosyl-glucoside	Flavones	C26 H28 O14	8239 5	208 42	104, 1078	1,20 7	6	Posi tive	564, 1481	75,9 4	569, 1275	1,11 4
Chrysoeriol 7-O-(6''-malonyl-apiosyl-glucoside)	Flavones	C30 H32 O18	1507 524	253 500	122, 0813	8,89 6	4	Posi tive	680, 1565	90,5 8	663, 1532	8,69 6
Apigenin 6,8-di-C-glucoside	Flavones	C27 H30 O15	7419 8	156 99	122, 0814	11,1 57	3	Posi tive	594, 1583	74,6 9	617, 1476	11,0 72
Apigenin 6,8-di-C-glucoside	Flavones	C27 H30 O15	1134 527	184 575	512, 276	10,8	5	Posi tive	594, 1583	99,3 9	617, 1476	10,6 17
Apigenin 6,8-di-C-glucoside	Flavones	C27 H30 O15	1820 56	338 97	122, 0814	10,2 05	4	Posi tive	594, 1582	92,3 4	617, 1474	10,1 22
Apigenin 6,8-di-C-glucoside	Flavones	C27 H30 O15	7802 2	118 90	498, 2603	9,22 9	3	Posi tive	594, 1582	75,5	595, 1654	9,11 2
Cirsimaritin	Flavones	C17 H14 O6	8117 5	150 83	122, 0813	17,8 01	3	Posi tive	314, 0795	73,8	297, 0761	17,6 45
Diosmin	Flavones	C28 H32 O15	1860 42	355 27	122, 0813	7,82 8	3	Posi tive	608, 1738	98,5 5	631, 1629	7,72 6
Biochanin A	Isoflavonoids	C16 H12 O5	4701 34	829 25	122, 0813	17,6 88	3	Posi tive	284, 0686	99,6 5	285, 0759	17,5 26
Luteolin 7-O-(2-apiosyl-glucoside)	Flavones	C26 H28 O15	1665 473	277 699	122, 0813	9,51	7	Posi tive	580, 1427	99,6 4	603, 132	9,33
3,7-Dimethylquercetin	Flavonols	C17 H14 O7	3738 67	529 14	122, 0812	3,07 4	4	Posi tive	330, 0739	85,5 2	353, 0632	2,97 5

6,8-Dihydroxykaempferol	Flavonols	C15 H12 O8	624554	48513	122,0813	8,221	2	Positive	320,0532	81,83	303,0499	8,004
Isorhamnetin	Flavonols	C16 H12 O7	3696820	586968	647,159	11,231	4	Positive	316,0585	99,02	317,0657	10,874
Isorhamnetin	Flavonols	C16 H12 O7	7288231	1247066	633,1434	9,865	4	Positive	316,0587	98,78	317,066	9,528
Isorhamnetin 3-O-galactoside	Flavonols	C22 H22 O12	5138453	632193	512,2759	11,033	7	Positive	478,1113	99,61	501,1006	10,656
Isorhamnetin 3-O-galactoside	Flavonols	C22 H22 O12	996716	171462	633,1434	9,746	5	Positive	478,111	99,67	479,1183	9,528
Isorhamnetin 3-O-galactoside	Flavonols	C22 H22 O12	667282	85034	498,2603	9,409	3	Positive	478,1108	96,91	479,1181	9,211
Isorhamnetin 3-O-glucoside 7-O-rhamnoside	Flavonols	C28 H32 O16	2477827	396332	512,2759	10,775	7	Positive	624,169	99,5	647,1583	10,696
Isorhamnetin 3-O-glucoside 7-O-rhamnoside	Flavonols	C28 H32 O16	3078903	398718	498,2603	9,389	4	Positive	624,1691	99,8	625,1765	9,211
Kaempferide	Flavonols	C16 H12 O6	108459	16774	122,0813	15,25	3	Positive	300,0635	77,72	301,0708	15,071
Kaempferol	Flavonols	C15 H10 O6	134943	19187	512,276	10,775	2	Positive	286,0479	84,42	287,0551	10,617
Kaempferol	Flavonols	C15 H10 O6	361666	62462	122,0813	9,488	2	Positive	286,0476	85,85	287,0549	9,31
Kaempferol	Flavonols	C15 H10 O6	272104	40684	498,2603	9,231	2	Positive	286,0476	84,92	287,0548	9,112
Kaempferol 3-O-(2''-rhamnosyl-6''-acetyl-galactoside) 7-O-rhamnoside	Flavonols	C35 H42 O20	688954	73103	122,0813	12,874	3	Positive	782,2287	93,78	787,2074	12,735
Kaempferol 3-O-(2''-rhamnosyl-galactoside) 7-O-rhamnoside	Flavonols	C33 H40 O19	456169	81022	498,2602	9,211	6	Positive	740,2144	87,75	741,2234	9,092
Kaempferol 3-O-(2''-rhamnosyl-galactoside) 7-O-rhamnoside	Flavonols	C33 H40 O19	559450	99636	122,0813	9,033	5	Positive	740,215	81,28	763,2052	8,934

6-Hydroxyluteolin 7-O-rhamnoside	Flavones	C21 H20 O11	190867	22444	512, 2759	10,835	3	Positive	448, 1001	74,43	471, 0893	10,617
6-Hydroxyluteolin 7-O-rhamnoside	Flavones	C21 H20 O11	153949	21795	122, 0813	10,379	3	Positive	448, 1001	73,22	471, 0894	10,201
Kaempferol 3-O-glucosyl-rhamnosyl-galactoside	Flavonols	C33 H40 O20	25475968	3479360	498, 2603	9,667	10	Positive	756, 2123	98,26	757, 2198	9,211
Kaempferol 3-O-glucosyl-rhamnosyl-galactoside	Flavonols	C33 H40 O20	425905	71721	122, 0813	8,241	5	Positive	756, 2111	95,19	757, 2185	8,103
Kaempferol 3-O-glucosyl-rhamnosyl-galactoside	Flavonols	C33 H40 O20	393401	70814	122, 0813	8,023	5	Positive	756, 2109	96,6	779, 2003	7,924
Kaempferol 3,7-O-diglucoside	Flavonols	C27 H30 O16	32431359	5036568	633, 1433	9,825	9	Positive	610, 1539	99,46	611, 1607	9,528
Kaempferol 3,7-O-diglucoside	Flavonols	C27 H30 O16	775445	109931	498, 2603	9,37	6	Positive	610, 1533	97,77	633, 142	9,271
Kaempferol 3-O-xylosyl-rutinoside	Flavonols	C32 H38 O19	1140687	193879	498, 2603	9,271	7	Positive	726, 2002	99,11	749, 1895	9,132
Myricetin 3-O-rhamnoside	Flavonols	C21 H20 O12	221438	31202	122, 0813	9,488	4	Positive	464, 0951	80,21	487, 0842	9,31
Myricetin 3-O-rhamnoside	Flavonols	C21 H20 O12	668491	113424	498, 2602	9,211	5	Positive	464, 095	99,13	487, 0843	9,073
Quercetin 3-O-(6	Flavonols	C30 H32 O15	123584	20892	122, 0813	12,854	2	Positive	632, 1759	70,13	655, 1652	12,735
Quercetin 3-O-arabinoside	Flavonols	C20 H18 O11	359959	58988	122, 0813	8,954	3	Positive	434, 0844	87,44	439, 0633	8,815
Quercetin 3-O-xylosyl-rutinoside	Flavonols	C32 H38 O20	1469449	243799	122, 0813	8,056	7	Positive	742, 1953	99,11	765, 1846	7,885
Quercetin 4'-O-glucoside	Flavonols	C21 H20 O7	145242	18696	122, 0813	8,063	3	Positive	384, 1199	96,27	366, 1088	7,865
6''-O-Acetyldaidzin	Isoflavonoids	C23 H22 O10	65941	11297	122, 0813	8,716	3	Positive	458, 1219	75,97	481, 1108	8,617

6''-O-Acetylgenistin	Isoflavonoids	C23 H22 O11	105585	19889	122, 0812	6,063	4	Posi tive	474, 1158	88,71	497, 1054	5,945
6''-O-Malonylgenistin	Isoflavonoids	C24 H22 O13	4680108	667036	512, 2759	10,993	4	Posi tive	518, 1038	88,84	501, 1006	10,656
Glycitin	Isoflavonoids	C22 H22 O10	50783	9531	122, 0813	9,092	2	Posi tive	446, 1215	72,75	451, 1002	9,033
1-Acetoxypinoresinol	Lignans	C22 H24 O8	422930	43602	104, 1079	1,411	7	Posi tive	416, 1473	86,2	439, 1368	1,134
Cyclolariciresinol	Lignans	C20 H24 O6	205432	15920	293, 2116	21,11	2	Posi tive	360, 1567	81,95	343, 1533	20,931
Dimethylmatairesinol	Lignans	C22 H26 O6	2909087	494502	122, 081	17,407	9	Posi tive	386, 1728	98,74	387, 18	17,17
Syringaresinol	Lignans	C22 H26 O8	54051	10985	122, 0813	7,984	3	Posi tive	418, 1645	85,1	423, 143	7,905
4-Vinylsyringol	Alkylmethoxyp henols	C10 H12 O3	821657	55872	365, 1061	21,304	2	Posi tive	180, 0786	87,39	163, 0753	20,912
4-Vinylsyringol	Alkylmethoxyp henols	C10 H12 O3	174596	28060	122, 0813	3,109	4	Posi tive	180, 0781	70,64	181, 0863	2,995
3-Methylcatechol	Alkylphenols	C7 H8 O2	1294640	210891	263, 0531	5,289	3	Posi tive	124, 0524	97,39	107, 049	5,034
3-Methylcatechol	Alkylphenols	C7 H8 O2	264729	89667	365, 1057	2,397	5	Posi tive	124, 0524	93,1	107, 049	2,381
3-Methylcatechol	Alkylphenols	C7 H8 O2	466337	19715	122, 0814	0,976	3	Posi tive	124, 0526	97,75	107, 0493	0,817
4-Ethylphenol	Alkylphenols	C8 H10 O	8238433	39146	738, 6626	33,344	3	Posi tive	122, 0733	97,85	105, 07	30,395
4-Vinylphenol	Alkylphenols	C8 H8 O	1165060	356557	166, 0868	2,48	3	Posi tive	120, 0577	87,74	103, 0544	2,421
5-Heneicosylresorcinol	Alkylphenols	C27 H48 O2	24787	17809	871, 5751	32,949	2	Posi tive	404, 3639	76,94	409, 3426	32,929
5-Nonadecenylresorcinol	Alkylphenols	C25 H42 O2	2349102	15500	738, 6626	33,701	5	Posi tive	374, 3188	80,49	357, 315	31,206

p-Anisaldehyde	Hydroxybenzaldehydes	C8 H8 O2	784443	102399	349,0897	5,192	3	Positive	136,0523	87,74	119,049	4,856
Vanillin	Hydroxybenzaldehydes	C8 H8 O3	163331	27848	122,0813	8,93	3	Positive	152,0476	87,5	153,0547	8,815
Vanillin	Hydroxybenzaldehydes	C8 H8 O3	111522	17749	122,0812	6,906	4	Positive	152,0475	85,48	153,0548	6,816
2,3-Dihydroxy-1-guaiacylpropanone	Hydroxybenzoketones	C10 H12 O5	784285	142757	379,1002	5,362	3	Positive	212,0684	99,23	195,065	5,192
3-Methoxyacetophenone	Hydroxybenzoketones	C9 H10 O2	5865013	39383	738,6626	33,186	2	Positive	150,068	86,41	151,0754	31,088
3-Methoxyacetophenone	Hydroxybenzoketones	C9 H10 O2	8904589	895192	268,1041	2,104	4	Positive	150,0678	99,3	151,0751	1,866
Sinapaldehyde	Hydroxycinnamaldehydes	C11 H12 O4	695038	96450	122,0813	7,113	3	Positive	208,0736	87,19	191,0703	6,855
Sinapaldehyde	Hydroxycinnamaldehydes	C11 H12 O4	268308	42598	122,0812	5,826	3	Positive	208,0736	87,02	191,0704	5,648
4-Hydroxycoumarin	Hydroxycoumarins	C9 H6 O3	1706508	187661	738,6627	29,603	3	Positive	162,0316	99,78	163,0389	29,207
4-Hydroxycoumarin	Hydroxycoumarins	C9 H6 O3	1543451	240382	149,0237	21,486	3	Positive	162,0318	99,8	163,039	21,327
Esculin	Hydroxycoumarins	C15 H16 O9	564955	37196	122,0813	2,39	2	Positive	340,0796	83,46	363,0687	2,045
Mellein	Hydroxycoumarins	C10 H10 O3	826128	130509	263,0531	5,232	4	Positive	178,0626	89,05	161,0594	5,034
1,4-Naphtoquinone	Naphtoquinones	C10 H6 O2	37246	6034	263,0531	5,133	2	Positive	158,037	75,4	159,0439	5,054
3,4-Dihydroxyphenylglycol	Other polyphenols	C8 H10 O4	173088	29284	122,0813	8,934	2	Positive	170,0579	87,5	153,0547	8,815
Arbutin	Other polyphenols	C12 H16 O7	350349	59817	122,0813	7,459	2	Positive	272,0899	86,02	277,0686	7,271

Phlorin	Other polyphenols	C12 H16 O8	8595582	1174463	104,108	1,589	7	Positive	288,0848	80,39	289,0922	1,094
Pyrogallol	Other polyphenols	C6 H6 O3	723494	116259	365,103	23,406	3	Positive	126,0315	97,6	149,0206	23,07
Carnosol	Phenolic terpenes	C20 H26 O4	161192	23161	365,106	20,793	2	Positive	330,1827	83,57	331,1901	20,674
Carvacrol	Phenolic terpenes	C10 H14 O	2778783	24293	738,6628	32,275	5	Positive	150,1046	85,23	133,1011	30,573
Hydroxytyrosol 4-O-glucoside	Tyrosols	C14 H20 O9	1591286	268888	122,0812	6,954	5	Positive	332,1112	91,21	337,0894	6,816
Ligstroside-aglycone	Tyrosols	C19 H21 O7	1825788	98486	122,0813	3,569	3	Positive	361,1297	92,77	366,1087	3,351
Oleoside 11-methylester	Tyrosols	C17 H24 O11	1814111	315177	122,0813	7,838	4	Positive	404,1319	99,76	409,1106	7,627
Oleoside dimethylester	Tyrosols	C18 H26 O11	445397	69351	122,0812	6,083	5	Positive	418,1472	98,55	441,1361	5,945
2-Hydroxybenzoic acid	Hydroxybenzoic acids	C7 H6 O3	238839	35484	738,6626	29,444	3	Positive	138,0318	84,24	121,0285	29,207
2-Hydroxybenzoic acid	Hydroxybenzoic acids	C7 H6 O3	520952	63520	149,0236	21,932	3	Positive	138,0319	86,07	121,0286	21,565
2-Hydroxybenzoic acid	Hydroxybenzoic acids	C7 H6 O3	696451	97975	149,0237	21,486	2	Positive	138,0318	87,69	121,0285	21,327
2-Hydroxybenzoic acid	Hydroxybenzoic acids	C7 H6 O3	158443	26851	122,081	3,015	3	Positive	138,032	82,53	139,0394	2,698
4-Hydroxybenzoic acid 4-O-glucoside	Hydroxybenzoic acids	C13 H16 O8	1618702	225388	122,0811	3,054	4	Positive	300,0844	99,64	323,0736	2,698
5-O-Galloylquinic acid	Hydroxybenzoic acids	C14 H16 O10	500744	61891	349,0897	5,034	2	Positive	344,0744	83,76	367,0637	4,836
Benzoic acid	Hydroxybenzoic acids	C7 H6 O2	124454	22288	122,0813	6,994	2	Positive	122,0366	77,09	105,0333	6,895

Benzoic acid	Hydroxybenzoic acids	C7 H6 O2	315859	53503	122,0812	6,816	3	Positive	122,0366	87,59	105,0333	6,717
Gallic acid	Hydroxybenzoic acids	C7 H6 O5	217358	16068	104,1073	1,47	2	Positive	170,0215	73,52	171,0288	1,272
Gallic acid ethyl ester	Hydroxybenzoic acids	C9 H10 O5	533269	45536	365,106	22,179	2	Positive	198,053	85,01	181,0497	21,585
Gallic acid ethyl ester	Hydroxybenzoic acids	C9 H10 O5	478286	64698	149,0237	21,486	2	Positive	198,0529	87,08	181,0496	21,327
Protocatechuic acid 4-O-glucoside	Hydroxybenzoic acids	C13 H16 O9	300649	54379	104,1075	1,272	2	Positive	316,0801	83,73	321,0586	1,154
1,2-Diferuloylgentiobiose	Hydroxycinnamic acids	C32 H38 O17	198112	33987	633,1433	9,706	3	Positive	694,2103	98,01	717,1994	9,568
1-Sinapoyl-2-feruloylgentiobiose	Hydroxycinnamic acids	C33 H40 O18	162403	24835	122,0813	8,499	3	Positive	724,2187	81,38	707,2152	8,4
24-Methylcholestanol ferulate	Hydroxycinnamic acids	C38 H58 O4	543449	78197	738,6615	27,484	6	Positive	578,4342	90,16	601,4213	27,187
24-Methylenecholestanol ferulate	Hydroxycinnamic acids	C38 H57 O4	439417	70289	738,6615	27,405	4	Positive	577,4278	71,24	600,4164	27,168
3,4-Diferuloylquinic acid	Hydroxycinnamic acids	C27 H28 O12	1652900	86873	122,0811	3,173	3	Positive	544,1607	75,84	527,1576	2,361
3-Feruloylquinic acid	Hydroxycinnamic acids	C17 H20 O9	308701	20867	122,0813	7,568	3	Positive	368,1125	73,63	373,0895	7,469
3-Feruloylquinic acid	Hydroxycinnamic acids	C17 H20 O9	543490	67676	122,0812	6,242	4	Positive	368,1114	83,03	391,1003	5,925
3-Sinapoylquinic acid	Hydroxycinnamic acids	C18 H22 O10	63063	15646	122,0813	7,246	3	Positive	398,1225	81,49	421,1101	7,192
3-Sinapoylquinic acid	Hydroxycinnamic acids	C18 H22 O10	240949	18106	268,1048	1,807	4	Positive	398,1209	89,42	399,1275	1,609
3-p-Coumaroylquinic acid	Hydroxycinnamic acids	C16 H18 O8	63904	13737	122,0813	12,458	2	Positive	338,1004	81,37	339,1076	12,359
3-p-Coumaroylquinic acid	Hydroxycinnamic acids	C16 H18 O8	517581	78710	513,1584	6,499	4	Positive	338,1002	98,45	339,1072	6,242

3-p-Coumaroylquinic acid	Hydroxycinnamic acids	C16 H18 O8	121913	24947	379,1002	5,291	3	Positive	338,1009	80,03	339,1072	5,173
3-p-Coumaroylquinic acid	Hydroxycinnamic acids	C16 H18 O8	826277	72836	104,1071	1,585	3	Positive	338,101	80,85	321,0975	1,352
5-5'-Dehydrodiferulic acid	Hydroxycinnamic acids	C20 H18 O8	55860	11114	122,0812	3,601	3	Positive	386,1011	70,03	369,098	3,549
Caffeoyl glucose	Hydroxycinnamic acids	C15 H18 O9	62723	16749	122,0812	5,526	2	Positive	342,0942	75,14	343,1016	5,47
Caffeoyl glucose	Hydroxycinnamic acids	C15 H18 O9	218373	36346	122,0813	4,46	4	Positive	342,0941	79,92	343,1012	4,341
Caffeoyl tartaric acid	Hydroxycinnamic acids	C13 H12 O9	172110	25158	104,1075	1,343	3	Positive	312,0484	74,95	335,0376	1,134
Cinnamic acid	Hydroxycinnamic acids	C9 H8 O2	214935	39350	122,0813	18,694	2	Positive	148,0519	85,96	149,0592	18,615
Feruloyl glucose	Hydroxycinnamic acids	C16 H20 O9	3122869	440757	122,0812	6,063	7	Positive	356,1107	99,21	379,1	5,766
Feruloyl glucose	Hydroxycinnamic acids	C16 H20 O9	12850133	2045760	379,1002	5,529	7	Positive	356,1108	99,22	379,1002	5,192
Hydroxycaffeic acid	Hydroxycinnamic acids	C9 H8 O5	4303096	590865	104,1083	1,728	3	Positive	196,0373	77,85	219,0269	1,055
Isoferulic acid	Hydroxycinnamic acids	C10 H10 O4	1958208	265525	513,1584	6,598	4	Positive	194,058	99,65	177,0547	6,242
Isoferulic acid	Hydroxycinnamic acids	C10 H10 O4	2895790	395843	263,053	5,45	7	Positive	194,0579	99,65	177,0546	5,192
m-Coumaric acid	Hydroxycinnamic acids	C9 H8 O3	315389	34550	104,1071	1,55	4	Positive	164,0471	78,06	165,0549	1,332
p-Coumaric acid 4-O-glucoside	Hydroxycinnamic acids	C15 H18 O8	20232278	2817227	349,0897	5,212	5	Positive	326,1005	99,17	349,0897	4,856
p-Coumaric acid 4-O-glucoside	Hydroxycinnamic acids	C15 H18 O8	3495023	294741	104,1072	1,589	6	Positive	326,1015	78,42	308,091	1,272

p-Coumaric acid ethyl ester	Hydroxycinna mic acids	C11 H12 O3	2349 03	461 20	263, 0531	5,07 4	2	Posi tive	192, 0786	87,0 1	193, 0859	4,99 4
Rosmarinic acid	Hydroxycinna mic acids	C18 H16 O8	5683 658	722 999	349, 0897	5,09 3	4	Posi tive	360, 0847	80,9 7	365, 0639	4,85 6
Sinapic acid	Hydroxycinna mic acids	C11 H12 O5	3497 24	463 24	122, 0813	6,66 6	5	Posi tive	224, 0685	87,1 4	207, 0652	6,49 9
Sitostanyl ferulate	Hydroxycinna mic acids	C39 H60 O5	1903 25	449 44	738, 6626	29,3 24	5	Posi tive	608, 4413	71,7 1	590, 4298	29,2 66
Verbascoside	Hydroxycinna mic acids	C29 H36 O15	4155 28	282 72	122, 0812	3,23 2	3	Posi tive	624, 2028	78,2 5	625, 2098	3,13 3
3,4-dihydroxyphenyl-2-oxypropanoic acid	Hydroxyphenyl propanoic acids	C9 H8 O4	1417 574	187 769	738, 6626	29,5 43	4	Posi tive	180, 0423	99,7 8	163, 0389	29,2 07
3,4-dihydroxyphenyl-2-oxypropanoic acid	Hydroxyphenyl propanoic acids	C9 H8 O4	2040 071	306 557	149, 0237	21,4 86	5	Posi tive	180, 0424	99,8	163, 039	21,3 27
Dihydro-p-coumaric acid	Hydroxyphenyl propanoic acids	C9 H10 O3	2149 35	393 50	122, 0813	18,6 94	2	Posi tive	166, 0625	85,9 6	149, 0592	18,6 15
Dihydro-p-coumaric acid	Hydroxyphenyl propanoic acids	C9 H10 O3	1394 46	417 51	166, 0868	2,48	2	Posi tive	166, 0627	71,4 1	149, 0598	2,44 1
Dihydrocaffeic acid	Hydroxyphenyl propanoic acids	C9 H10 O4	5084 22	784 25	349, 0897	5,01 4	3	Posi tive	182, 0579	87,2	165, 0545	4,85 6
Dihydrocaffeic acid	Hydroxyphenyl propanoic acids	C9 H10 O4	2480 74	274 50	104, 1071	1,51	2	Posi tive	182, 0578	78,0 6	165, 0549	1,33 2
d-Viniferin	Stilbenes	C28 H22 O6	8741 9	122 75	122, 0812	6,93 5	2	Posi tive	454, 1403	75,9	454, 1399	6,83 6
